# Regorafenib and Ruthenium Complex Combination Inhibit Cancer Cell Growth by Targeting PI3K/AKT/ERK Signalling in Colorectal Cancer Cells

**DOI:** 10.3390/ijms24010686

**Published:** 2022-12-30

**Authors:** Deepu Sharma, Fayyaz Rasool, Manjri Bharti, Komal M. Vyas, Sri Krishna Jayadev Magani

**Affiliations:** 1 Department of Life Sciences, Shiv Nadar Institution of Eminence Deemed to Be University, G B Nagar Dist., Dadri 201314, UP, India; 2 Department of Chemistry, Sardar Patel University, Vallabh Vidyanagar 388120, GJ, India

**Keywords:** ruthenium, regorafenib, combination therapy, synergism, PI3K/Akt/ERK signalling

## Abstract

Cancer is one of the leading cause of lethality worldwide, CRC being the third most common cancer reported worldwide, with 1.85 million cases and 850,000 deaths annually. As in all other cancers, kinases are one of the major enzymes that play an essential role in the incidence and progression of CRC. Thus, using multi-kinase inhibitors is one of the therapeutic strategies used to counter advanced-stage CRC. Regorafenib is an FDA-approved drug in the third-line therapy of refractory metastatic colorectal cancer. Acquired resistance to cancers and higher toxicity of these drugs are disadvantages to the patients. To counter this, combination therapy is used as a strategy where a minimal dose of drugs can be used to get a higher efficacy and reduce drug resistance development. Ruthenium-based compounds are observed to be a potential alternative to platinum-based drugs due to their significant safety and effectiveness. Formerly, our lab reported Ru-1, a ruthenium-based compound, for its anticancer activity against multiple cancer cells, such as HepG2, HCT116, and MCF7. This study evaluates Ru-1′s activity against regorafenib-resistant HCT116 cells and as a combination therapeutic with regorafenib. Meanwhile, the mechanism of the effect of Ru-1 alone and with regorafenib as a combination is still unknown. In this study, we tested a drug combination (Ru-1 and regorafenib) against a panel of HT29, HCT116, and regorafenib-resistant HCT116 cells. The combination showed a synergistic inhibitory activity. Several mechanisms underlying these numerous synergistic activities, such as anti-proliferative efficacy, indicated that the combination exhibited potent cytotoxicity and enhanced apoptosis induction. Disruption of mitochondrial membrane potential increased intracellular ROS levels and decreased migratory cell properties were observed. The combination exhibited its activity by regulating PI3K/Akt and p38 MAP kinase signalling. This indicates that the combination of REG/Ru-1 targets cancer cells by modulating the PI3K/Akt and ERK signalling.

## 1. Introduction

Cancer is the second largest cause of mortality worldwide after cardiovascular disease. Colorectal cancer (CRC) is the third most fatal malignancy, with more than 1.85 million cases and 850,000 deaths registered annually [1]. Chemotherapy is one of the standard treatment practices for all cancers, including CRC. Though the current regimen of chemotherapeutics is promising, acquired resistance and cellular heterogeneity in cancers is a major challenge. Therefore, there is an increasing demand for new therapeutics [2,3]. Thus, to avoid this situation, combination therapy of two different drugs with different mechanism of action is a common practice at present. The advantages of combination therapy showing synergistic effects include reduction in toxicity, dosage, and drug resistance. Thus, combination therapy is preferred against various diseases, including cancers [4].

The advent of platinum-based drugs is a breakthrough in cancer therapy. These platinum-based metallodrugs are potently effective against many cancers [5]. However, platinum-derived compounds show secondary complications, such as neurotoxicity, nephrotoxicity, myelotoxicity, drug resistance, and hair loss [6,7]. These drawbacks make the researcher ponder developing better alternative metallodrugs to platinum-based compounds. Ruthenium complexes have been identified, showing remarkable anticancer properties against several cancerous cell lines and exhibiting synergistic effects when combined with other anticancer drugs [8]. These compounds could be promising alternatives to platinum-based compounds due to their improved tumour selectivity, variable oxidation state, similarity in ligand exchange kinetics, and reduced toxicity toward normal tissue [9,10,11]. Various ruthenium complexes, depending on their molecular structure, were shown to induce apoptosis through the endoplasmic reticulum stress pathway, mitochondria-mediated pathway, and death-receptor-mediated pathways, depending on the ligands and tumour sites [12,13,14,15,16]. Some of these complexes act as theragnostic agents, radiosensitisers, and photodynamic and photothermal therapy agents [17,18]. The multi-functionality of this compound makes it feasible to be used with other different therapies, such as radio-targeted therapy and nanotechnology [19,20].

Our previous study reported on Ru-1, a novel arene ruthenium (II) tetrazolate compound with potent anticancer properties against multiple cancer cells, such as HepG2, HCT116, and MCF7. Ru-1 showed even higher efficiency than Cisplatin [21]. Given the promising therapeutic effect of Ru-1, it is conceivable that the combination of Ru-1 and regorafenib is potent against colorectal cancer cells. Regorafenib is an FDA-approved drug used as a third-line therapeutic for refractory metastatic colorectal cancer. It is a multi-kinase inhibitor which targets VEGFR-1, -2, -3, FGFR-1, PDGFR-β, RAF-1, BRAF, RET, KIT, TIE-2, and p38 MAP kinase [22]. Even with the benefits of regorafenib on survival in mCRC patients, its overall clinical effectiveness is still limited. Developing efficient approaches for mCRC treatment is a critical need. In fact, combination chemotherapy is a potential propitious approach to intensify the antitumour activity, overcoming resistance to cancer treatments and extending the disease-free state and survival. Considering this, we evaluated regorafenib and Ru-1 combination on human colon cancer cell lines, and effects on downstream signalling caused by this combination were investigated. The combinatorial efficacy is assayed in regorafenib-resistant (REG-HCT116-R) and regorafenib-sensitive HCT116 and HT-29 cell lines. This study focuses on understanding the combinatorial efficacy of Ru-1 and regorafenib, calculating the CI index, and looking for the mechanism of action of these drugs on cells. The cytotoxicity, ROS generation capabilities, and molecular alteration in signalling pathways, such as PI3K/Akt, P38 MAP kinase, and ERK signalling were evaluated.

## 2. Results

### 2.1. Cytotoxicity of Regorafenib-Ruthenium Combination on HCT116, HT29, and REG-HCT116-R Cell Lines

**Figure 1:**
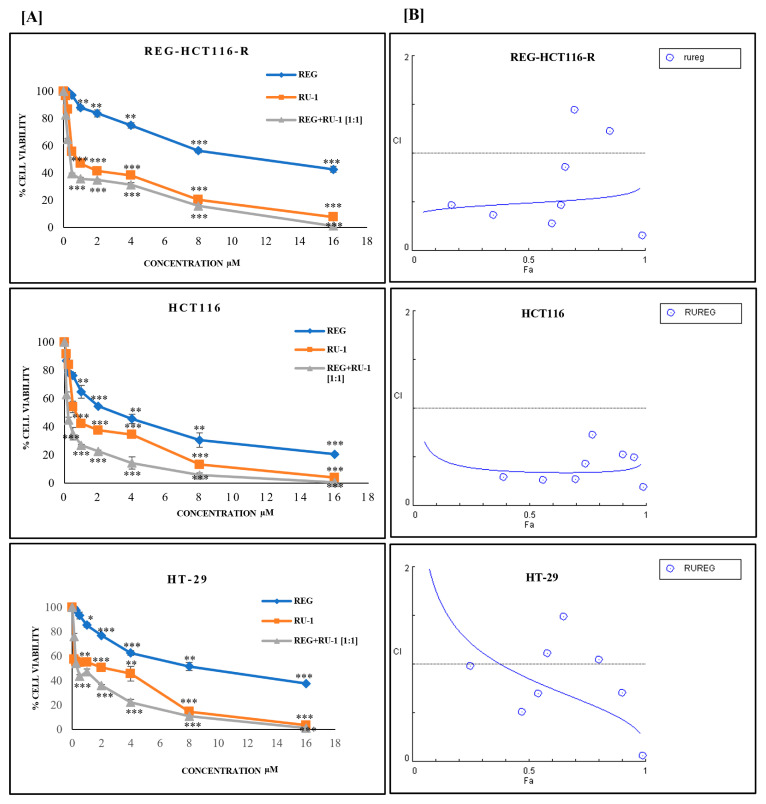
The cytotoxicity of the Reg/Ru-1 combination in different colon cancer cells—HCT116, REG-R-HCT116, and HT29—and the combination index. (**A**) Cell viability under the treatment with Reg/Ru-1 and determined by MTT assay. The X-axis denotes the drug’s concentration, and the Y-axis denotes the percentage of cell viability. IC_50_ values evaluated from full dose–response curve (drug concentration inducing a 50% reduction of cell number in comparison with untreated control cells cultured in parallel). Values given are means ± standard deviation of experiments performed in triplicates, and statistical significance was calculated using two-way ANOVA, * *p* < 0.05, ** *p* < 0.01, *** *p* < 0.001; (**B**) Plots showing combination index (CI) and fraction affected (Fa denotes the fraction of viable cells) of the Reg/Ru-1 combinations in colorectal cancer cell lines. Synergism is expressed by the combination index (CI) according to Chou and Talalay using Compusyn Software ver.1.0 (Combosyn, lnc., Paramus, NJ, 07652, USA).

The cytotoxicity of the drugs—regorafenib, Ru-1, and their combinations—were studied against HCT116, regorafenib-resistant HCT116, and HT29 after 48 h of incubation (Figure 1A). For cell lines HCT116, REG-HCT116-R, and HT29, there was significant effects of drug treatment: (HCT116, F _2,54_ = 921.5, *p* < 0.0001), (REG-HCT116-R, F _2,54_ = 4867, *p* < 0.0001) and (HT29, F _2,54_ = 2213, *p* < 0.0001); drug concentration: (HCT116, F _8,54_ = 1467, *p* < 0.0001), (REG-HCT116-R, F _8,54_ = 3301, *p* < 0.0001), and (HT29, F _8,54_ = 1429, *p* < 0.0001); and their interactions: (HCT116, F _16,54_ = 39.13, *p* < 0.0001), (REG-HCT116-R, F _16,54_ = 159, *p* < 0.0001), and (HT29, F _16,54_ = 65.65, *p* < 0.0001) on the cell viability. Pairwise comparison showed that different drug treatments, drug concentration, and their interactions had significant difference in cell viability (Supplementary Table S1). The combination of Reg/Ru-1 showed lower IC_50_ values of 0.4 µM, 1.2 µM, and 0.9 µM in HCT116, REG-HCT116-R, and HT29 cell lines, respectively (Table 1). For the drug combination, regorafenib co-treatment with Ru-1 exhibited synergistic effects in all cell lines analysed with IC_50_ 0.4 µM for HCT116 and 0.9 µM for HT29. Notably, the Reg/Ru-1 drug combination showed synergistic activity in regorafenib-resistant cells with IC_50_ 1.2 µM. In Figure 1B and Table 2, the CI index values reported that the combination herein was noticed to be less than 1, indicating the combination’s synergistic effect.

We also determined the possible impact of this drug combination on clonogenic growth to further examine its effects on cell growth. HCT116 and REG-HCT116-R cells were used for these studies, and both cell types were exposed to varying doses of both drugs for 24 h. Cell clones were fixed, stained, and counted after an additional 14 days. As in Supplementary Figure S1D,E, both the HCT116 and REG-HCT116-R cell lines’ clonogenic growth was significantly inhibited by this combination of regorafenib and Ru-1. This further confirmed the effective inhibitory activity of Ru-1 in combination with regorafenib.

### 2.2. Regorafenib–Ruthenium Combination-Induced Cell Death

A propidium iodide dye exclusion test was used to count the amount of live and dead cells. Compared with control cells, the data showed that the drug treatment caused significant cell death. As shown in Supplementary Figure S1A–C, compared with untreated control cells and individual drug-treated cells, cells treated with the combination of Reg/Ru-1 displayed a higher positivity for red fluorescence. A higher percentage of cell death was seen in cells treated with a combination of the two drugs in all the cell lines. This result further confirmed the cytotoxicity of the Reg/Ru-1 combination established by the MTT assay.

**Figure 2:**
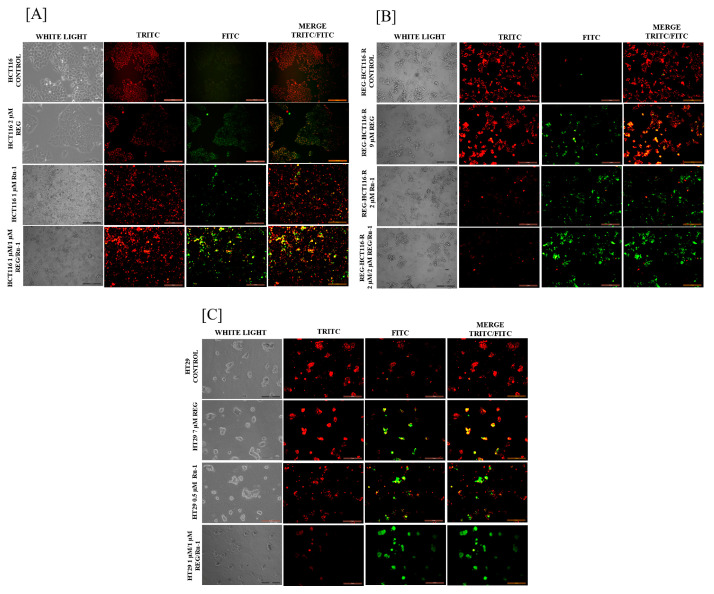
Mitochondrial membrane potential loss analysis was carried out by JC-1 staining on (**A**) HCT116 cells, (**B**) REG-R-HCT116 cells, and (**C**) HT29 cells treated with Ru-1 alone, regorafenib alone, and Reg/Ru-1 combination imaged under a fluorescence microscope with 20× magnification. Scale bar 200 µm. Red—Intact mitochondria and Green—Potential lost mitochondria.

Regorafenib–ruthenium combination treatment increases mitochondrial membrane potential loss. A loss of mitochondrial membrane potential characterises apoptosis. An essential aspect of mitochondrial function that can be utilised to gauge the health of a cell is membrane potential. As shown in Figure 2, the untreated control cells showed more excellent red fluorescence, suggesting intact mitochondrial membrane potential; the drug-treated cells showed an increase in green fluorescence, indicating membrane integrity loss. The results showed significant membrane potential loss comparing drug-treated cells with untreated cells.

**Figure 3:**
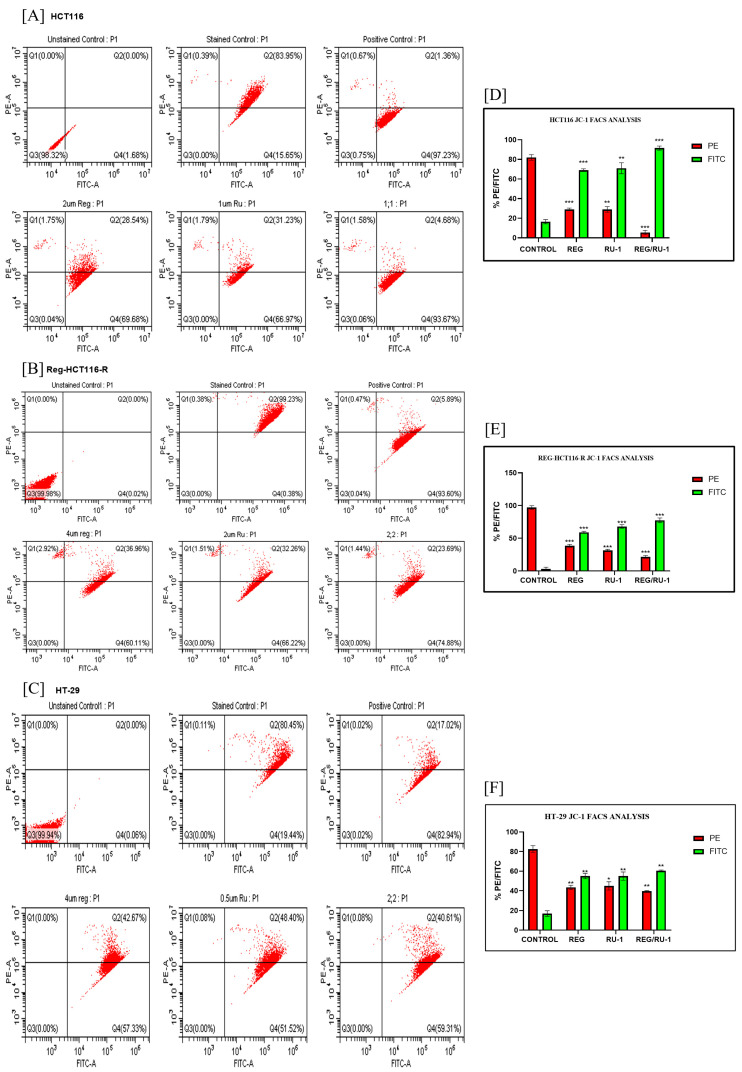
Quantitative analysis of mitochondrial membrane potential loss (MMP) assayed by flow cytometry: (**A**) HCT116 cells, (**B**) REG-R-HCT116 cells, and (**C**) HT29 cells treated with Ru-1 alone, regorafenib alone, and Reg/Ru-1 combination. (**D**–**F**) The bar graph shows the percentage population of red and green fluorescence-positive cells of HCT116, REG-R-HCT116, and HT29, respectively. * *p* < 0.05, ** *p* < 0.01, *** *p* < 0.001.

The above results were then quantified by flow cytometry. For FACS analysis of the decrease in membrane potential, HCT116, regorafenib-resistant HCT116, and HT29 cells were treated with Ru-1 alone, regorafenib alone, and in a combination of both; the drugs and were stained with JC-1 dye. As shown in Figure 3, cells with an undamaged mitochondrial membrane exhibited higher levels of PE positivity, whereas those treated with the drug exhibited dose-dependent FITC positivity. The percentage of green fluorescence in regorafenib alone and Ru-1 alone treated cells was observed in HCT116 (69.68%) with 2 µM Reg treatment, REG-HCT116-R (60%) with 4 µM Reg treatment, HT29 (57.33%) with 4 µM Reg treatment, HCT116 (66.97%) with 1 µM RU-1, REG-HCT116-R (66.22%) with 2 µM Ru-1, and HT29 (51.52%) with 0.5 µM Ru-1, which was found to be increased in combination-treated cells at 1:1 concentration in all three cell lines, HCT116 (93.67%), REG-HCT116-R (74.88%), and HT29 (59.31%), respectively. The green and red population changes in treated and control cells are depicted in the bar diagram. CCCP treatment is used as a positive control for mitochondrial membrane depolarization. In cells treated with Reg/Ru-1, a sharp rise in the proportion of green-stained cells to almost 90% and a fall in red-stained cells suggest mitochondrial membrane potential loss, confirming the disruption of membrane integrity.

### 2.3. Reactive Oxygen Species Generation Increases in Reg/Ru-1-Treated Cells

**Figure 4:**
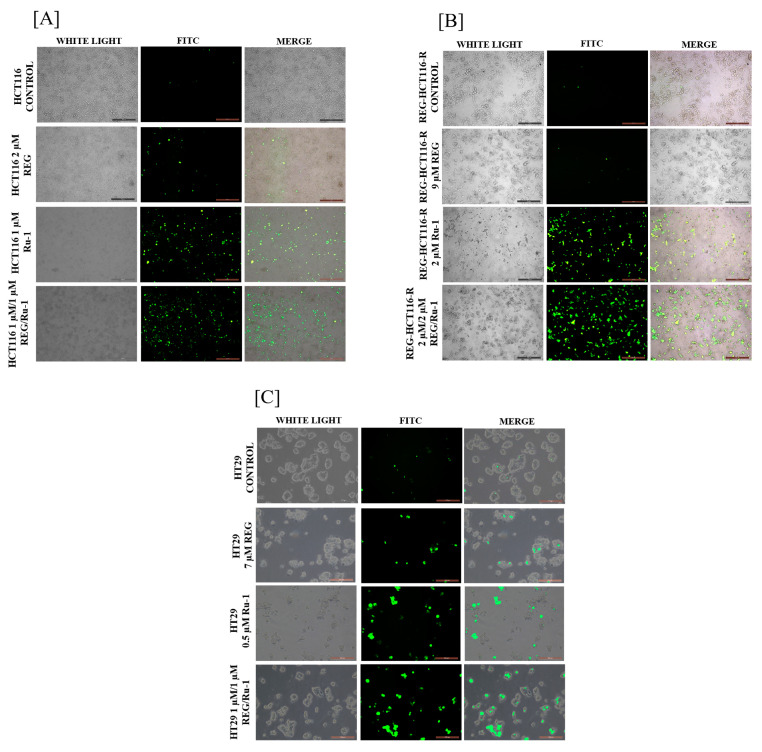
ROS generation analysis by DCHF-DA staining on (**A**) HCT-116, (**B**) REG-HCT116-R, and (**C**) HT29 cells treated with Ru-1 alone, regorafenib alone, or in combination. Imaged in a fluorescent microscope at 10X magnification (HCT116 and REG-HCT116-R). Scale bar 100 µm and 20× magnification was used for HT29 cells imaging. Scale bar 200 µm. The green population indicates positivity for ROS.

Key biological processes, such as cell differentiation, growth, and death produce reactive oxygen species, such as superoxides and peroxides. If present in large amounts, these species can stress cells and cause them to die through apoptosis and autophagy. Superoxide dismutase (SOD), glutathione peroxidase, and glutathione (GSH), free radical scavengers that protect cells by converting harmful free radicals to safe ones, are upregulated by the cellular homeostasis machinery to balance this. A fluorescent probe called DCFH-DA is utilised to find intracellular oxidative stress. In the presence of ROS, DCFH-DA is hydrolysed to DCFH and kept intracellularly. Fluorescent dichlorofluorescein, which fluoresces green, is produced when DCFH is oxidised [23] As shown in Figure 4, treated HCT116, regorafenib-resistant HCT116, and HT29 cells exhibited increased green fluorescence compared with untreated cells, indicating ROS generation. Green fluorescence was highest in combination Reg/Ru-1 treated cells.

### 2.4. Drug-Combination-Treated Cells Exhibited Effective Inhibition in Wound-Healing and Cell Migratory Activity

**Figure 5:**
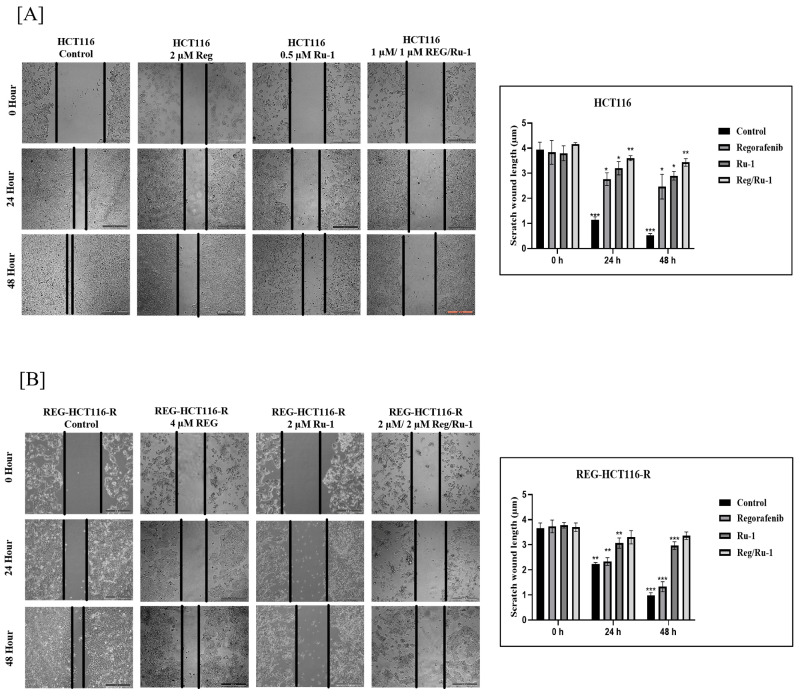
Cell migration inhibition of Ru-1, regorafenib, and Reg/Ru-1 combination-treated cells by wound-healing assay. (**A**) HCT116 and (**B**) REG-HCT116-R cells were treated with drugs and wound-healing capability of cells was investigated with that of control cells after incubating for 24 and 48 h. Scale bar 100 µm. The graphical representation shows scratch wound length in µm at 0 h, 24 h, and 48 h incubation after treatment with single agents and in combination. * *p* < 0.05, ** *p* < 0.01, *** *p* < 0.001.

The wound-healing assay is a simple, reliable, and indicative approach to determine cell migration, cell–cell interaction, and wound-healing capabilities that indicate cells’ metastatic properties. As seen in Figure 5A,B, HCT116 and REG-HCT116-R control cells showed effective migration and wound-healing ability, close to 95% by 48 h. In contrast, cells treated with drugs exhibited no or insignificant wound-healing activity. A significant decrease in wound-healing activity was seen in cells treated with the Reg/Ru-1 combination, thereby demonstrating the anti-metastatic capabilities of the combination.

### 2.5. Change in Apoptosis-Associated Genes and Protein Expression in Reg/Ru-1-Treated Cells

**Figure 6:**
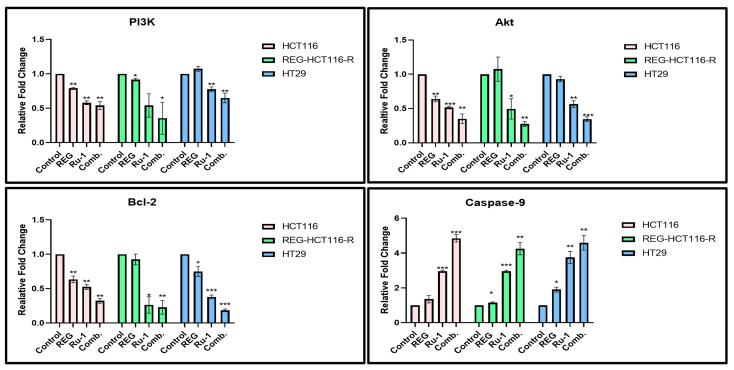
Graphical representation of gene expression of *PI3K, Akt, Bcl-2*, and *caspase-9*. Down-regulation was seen in the case of *PI3K, Akt* and *Bcl-2*, upon treatment of cells with IC_50_ concentration of Ru-1 alone, Reg alone, or in combinations of both the drugs in all three cell lines HCT116, REG- HCT116-R, and HT29, whereas up-regulation was seen in case of *caspase-9*. * *p* < 0.05, ** *p* < 0.01, *** *p* < 0.001.

Real-time qPCR was used to assess the gene expression profiles of genes associated with apoptosis in cells treated with Ru-1, regorafenib, and Reg/Ru-1 combination. The transcriptional expression of mRNAs of apoptosis-related genes *Bcl-2*, *caspase-9*, *PI3K*, and *Akt* were analysed for apoptotic induction. One of the characteristics of cancer cells was said to be avoiding apoptosis. Compared with control cells, the anti-apoptotic gene *Bcl-2* showed decreased expression (Figure 6). All three cell lines showed a reduction in expression, supporting the induction of apoptosis. *Caspase-9* is a crucial player in the intrinsic or mitochondrial-mediated apoptotic pathway. This expression was reported to be associated with chemotherapy, stress, and radiation [24]. Comparing drug-treated cells with control cells, *caspase-9* expression was higher in the drug-treated cells. The drug-treated cells of all three cell lines showed a multi-fold change in *caspase-9* expression.

The PI3K pathway’s activation aids in the growth of malignancies. Both cellular proliferation and cell death can be affected by PI3K inhibition [25,26]. A decrease in PI3K expression was noticed in drug-treated cells compared with the control population. However, the proportion of effect was least in the HT29 cell line. This could be attributed to the wild-type PIK3CA in HT29 cells in comparison with the mutant-activated PIK3CA in HCT116 cells. This mutation leads to overexpression of PI3K/AKT signalling in HCT116 cells. Whereas the HT29 cells have a wild-type PIK3CA, the expression change in drug-treated HT29 cells is not as prominent as in HCT116 [27]. Akt1, also known as Akt kinase, is a serine/threonine-protein kinase that regulates several signalling downstream pathways, including angiogenesis, cell growth, survival, and proliferation [28,29]. A similar trend of decreased expression of this gene in drug-treated cells further confirms our apoptosis induction by these drug combinations.

**Figure 7:**
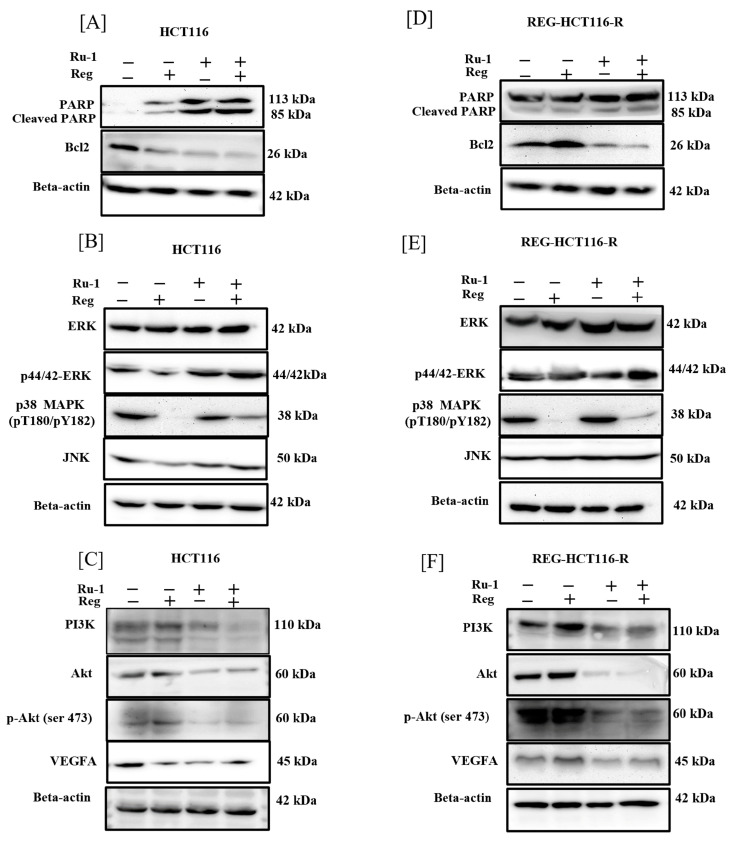
Western blot for ERK, p44/42-ERK1/2, p38, JNK, VEGFA, Akt, p-Akt (Ser 473), PI3K, cleaved PARP, and Bcl-2. HCT116 and REG-HCT116-R cells were treated with IC_50_ concentration of Ru-1 alone, regorafenib alone, or in combination with a 1:1 ratio for 24 h, and western blot analysis was conducted with the indicated antibodies (p-represents phosphorylation of proteins). (**A**) Expression of cleaved PARP and Bcl-2 in HCT116 cells. (**B**) Expression of ERK, p-ERK, p38, and JNK in HCT116 cells. (**C**) Expression of Akt, P-Akt, PI3K, and VEGFA in HCT116 cells. (**D**) Expression of cleaved PARP and Bcl-2 in REG-HCT116-R cells. (**E**) Expression of ERK, p-ERK, p38, and JNK in REG-HCT116-R cells. (**F**) Expression of Akt, P-Akt, PI3K, and VEGFA in REG-HCT116-R cells.

We used western blotting to evaluate the expression of cleaved PARP and Bcl-2 to investigate additional apoptotic markers. Regorafenib (2 μM) or Ru-1 (1 μM) alone had minimal impact on the induction of cleaved PARP expression, while the two drugs’ combination had a considerable impact on PARP cleavage in HCT116 and REG-HCT116-R cells. Additionally, we noticed that drug combination decreased the expression of Bcl-2. It has been proven that Bcl-2 has potent anti-apoptotic properties by maintaining the integrity of the mitochondrial membrane, preventing the oligomerisation and translocation of BAX and BAK to the mitochondrial membrane and preventing the formation of apoptosomes, which increases the number of cancer cells by preventing cell death and promotes resistance to chemotherapy in cancer cells. (Figure 7A,D)

### 2.6. Reg/Ru-1 Combination Treatment Inhibits the PI3K/Akt Signalling and Activates the ERK-Mediated Cell–Death Pathway

In human CRC, the RAS/ERK/RAF, NF-κB, and PI3K/Akt/mTOR signalling pathways are well-known mechanisms for survival. It has been demonstrated that unregulated activation of each pathway promotes cell proliferation and the emergence of cellular drug resistance. Regorafenib, a multi-kinase inhibitor, inhibits several numbers of protein kinases implicated in tumour growth (KIT, RET, RAF-1, and BRAF p38 MAP kinase), metastasis (PDGFR- and FGFR1), and tumour angiogenesis (VEGFR-1, -2, and -3) [30]. However, regorafenib has been found to be a marginally effective inhibitor of the PI3K/Akt pathway. Consequently, their activation could result in regorafenib resistance, and we also found activation of the PI3K/Akt pathway in the proteome profile of the regorafenib-resistant HCT116 cell line model (data not shown) [31,32,33]. Human malignancies frequently exhibit abnormal activation of the intracellular signalling pathway MAP–kinase [34]. Regorafenib, a multi-kinase inhibitor, also targets MAP kinase p38, thereby blocking the downstream signalling. The same was observed in both HCT116 and regorafenib-resistant HCT116 cells [34,35]. However, ruthenium compounds are known to cause oxidative stress [36], a well-reported factor to activate the p38 MAP kinase signalling, causing G2/M phase cell cycle arrest [37]. Hence, we further investigated the potential effect of regorafenib on Ru-1-mediated p38 activation. As shown in Figure 7B,E, treatment with Ru-1 activates p38 phosphorylation, which was inhibited via combined treatment with regorafenib in HCT116 cells. In contrast, in regorafenib-resistant HCT116 cells, we could observe a decrease in expression but not total loss of expression. Further, to confirm our results obtained by qPCR, we performed immunoblotting of pAkt, PI3K, p-ERK, p38 MAP kinase and total Akt, JNK, and ERK proteins (Figure 7). These proteins are associated with cell proliferation, survival, and cell death by apoptosis pathways. Regorafenib was shown to inhibit PI3K/Akt weakly compared with Ru-1, which showed a higher inhibition (Figure 7C,F). The combination of two agents efficiently inhibited the PI3K/Akt signalling. In addition, treatment with Reg had little effect on p-ERK expression in HCT116 cells, and no such effect was seen on regorafenib-resistant HCT116 cells. Increased levels of p-ERK were observed in Ru-1 and drug combinations. We noticed an increase in JNK expression in Ru-1 alone and combination-treated cells. In contrast, regorafenib alone has shown a minimal effect on JNK expression in HCT116, and no such significant impact on the expression of JNK was seen in regorafenib-resistant HCT116 cells. We could also see a decreased expression of VEGFA, an angiogenesis marker, in combination with treatment in both cell lines. A reduction in anti-apoptotic Bcl-2 and increased expression of PARP cleavage were recorded in drug-combination-treated cells. This further confirms the apoptosis induction capabilities of the drugs and their combination. As Akt is a kinase, and its activity is regulated by phosphorylation, we checked the levels of phosphorylated Akt, and we could see a decrease in the expression of phosphorylated Akt, further supporting the death-induction capabilities of the drugs [38]. Apoptosis induction is associated with ERK [39]. We noticed an increase in the expression of ERK and its phosphorylation, thereby further supporting our data on apoptosis induction by the drugs and their combination. The densitometric analysis data is attached as supplementary Figure S2.

## 3. Discussion

To conclude, this study is focused on evaluating the combinatorial therapeutic efficacy of ruthenium-based metallodrug in combination with regorafenib, an anti-metastatic drug used for mCRC (metastatic colorectal cancer) treatment. This study is the first of its kind reporting the combination of ruthenium compound with regorafenib-on-regorafenib-resistant colorectal cancer cells. In this study, we could observe that both the molecules exhibited cytotoxicity individually but showed a synergistic effect in combination. The IC_50_ values of combination are lower and statistically significant. The CI values indicated the synergistic effect of these drugs in combination.

**Figure 8:**
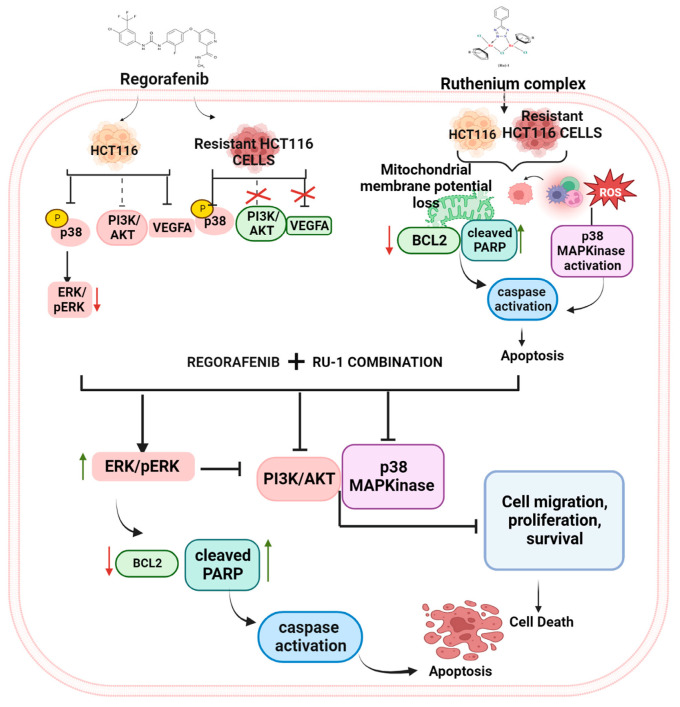
Image showing a representation of signaling pathways affected by treatment of Reg/Ru-1 combination in colorectal cancer cells.

Further, the PI exclusion experiments and the mitochondrial membrane potential experiments confirmed the induction of apoptosis by these drugs. Our data indicate a significant increase in ROS generation as well. The increased expression of *caspase-9*, a pro-apoptotic gene, and decreased expression of *Bcl-2*, an anti-apoptotic gene, further confirm the apoptotic induction capabilities of the drugs and their combination. The reduced expression of pro-survival Akt and PI3K reconfirm the anticancer properties of the drugs. The regorafenib-resistant cells showed an increased expression of the Akt pathway (data not shown here). The treatment with the ruthenium compound exhibited a decrease in Akt and phosphorylated Akt, thereby sensitising the cells to regorafenib. The increased expression of ERK and phosphorylated ERK further supports apoptosis by these drugs. As shown in Figure 8, we hypothesise that the apoptosis is through ROS-mediated stress leading to ERK phosphorylation, which inhibits the PI3K/Akt pathway and induces apoptosis through the intrinsic pathway. Our data is in line with earlier reports [40].

In our study, the regorafenib-resistant HCT116 cells generated in the lab showed a higher expression of Akt (data not shown here), and there are earlier reports of regorafenib as a weak inhibitor of PI3K/Akt signalling [41]. Hence, we checked the Akt inhibition by the ruthenium complex alone and in combination with regorafenib. Ruthenium compound exhibited an inhibiting effect on the Akt signalling pathway. The combination was shown to be more cytotoxic when compared with the individual drug molecules, thereby re-sensitising the resistant cells to regorafenib. The earlier reports of synergistic activity of ruthenium compounds in combination with other chemotherapeutic drugs, such as sorafenib, paclitaxel, doxorubicin, and cisplatin, supported the inhibition of multiple signalling pathways involved in cancer progression [42]. The reg-alone treatment enhanced Akt’s phosphorylation in the regorafenib-resistant HCT116 cell line, which was significantly suppressed by the combined treatment with Ru-1. In CRC cells, the combination of Ru-1 and regorafenib dramatically reduced pAkt, PI3K, p-ERK, p38 MAP kinase, and total Akt, JNK, ERK, and VEGFA.

On the other hand, treatment with Ru-1 enhanced the ERK phosphorylation in HCT116, and REG-HCT116-R cells were consistent with the earlier report [39,43]. However, ERK was shown to be a target of regorafenib. This was further confirmed by the expression profile of p38, which is the upstream effector molecule of ERK. Regorafenib was shown to inhibit p38 expression, whereas it was noticed that Ru-1 alone was shown to activate p38. However, in combination, ruthenium-based compounds are known to generate intracellular reactive oxygen species and trigger apoptosis through mitochondrial pathways. Here, our results demonstrated that regorafenib mediates the reduction of Ru-1-induced phosphorylation of p38 MAP kinase more efficiently. p38 is a stress-responsive kinase with multiple downstream targets compared with other MAP kinases, and it is also involved in cell death pathways [44]. Various reports suggested that activation of p38 MAP kinase by stress stimuli may not always result in cell death but may also support cell survival through G2/M cell cycle checkpoints and DNA damage repair pathways activation [37,45]. This confirms our results showing that regorafenib treatment suppresses the Ru-1-induced activation of p38 signalling.

The strategy of combination therapy is used to target the heterogeneity in tumours. The superiority of combination therapy is attributed to its ability to simultaneously target multiple pathways, thereby affecting the adaptive property of cancer cells for both drugs simultaneously. This would decrease the chance of drug resistance development and the recurrence of cancers. In our study, regorafenib targets tyrosine kinases essential for cancer survival and progression and ruthenium-induced apoptosis by ROS generation. The exact mechanism and pathways the ruthenium complex targets are yet to be identified. We could conclude that this combination is very effective in vitro and was able to re-sensitize the regorafenib-resistant HCT116 cells as well. The IC_50_ concentration of the combination indicates reduced drug quantities are required. Recent studies showed that ruthenium-based compounds showed more significant antitumor activity in cellular and in vivo models. In addition, studies reported that simultaneous use of Ru-1 with sorafenib potentiates the effectiveness of sorafenib [46]. However, the safety, efficacy, and mode of action of Reg/Ru-1 combination treatment need to be further determined by in vivo experiments. Additionally, the in vivo effect of these drugs combination needs a thorough scientific evaluation.

## 4. Materials and Methods

### 4.1. Materials

Ruthenium (Ru-1) with molecular weight 722 Dalton was dissolved in DMSO, and a 10 mM stock solution was prepared and synthesised as in our previous study [21]. Regorafenib (BAY 73-4506) was ordered from selleckchem.com in the United States in a concentration of 10 mM. To maintain the DMSO concentration below 0.5% during experimental studies, further working dilutions were prepared, which are not toxic for cells. Fetal bovine serum (FBS) and DMEM medium were obtained from Gibco (Thermo Fisher Scientific, Mumbai, India). Propidium iodide was purchased from HI Media (HI media labs, Mumbai, India), 3-(4,5-dimethylthiazol-2-yl) −2,5-diphenyl tetrazolium bromide (MTT), DCFH-DA, JC-1 staining kits were purchased from G-BIOSCIENCES (Noida, India). Molecular structures of Ru-1 and regorafenib are provided as Supplementary Data.

### 4.2. Cell Culture and Media

Dulbecco’s modified Eagle medium (DMEM) (GIBCO Thermo fisher Scientific, Mumbai, India, REF 11995-065) with 10% FBS (GIBCO Thermo fisher Scientific, Mumbai, India REF 10270-106) and 1% Penicillin/streptomycin antibiotics (HIMEDIA, REF A001-100ML) was used to grow HCT116 (colon cancer).This cell line was reported to have wild-type p53, BRAF, and PTEN, whereas the KRAS and PIK3CA are shown to be mutated REG-HCT116-R (regorafenib-resistant); HT-29 (colon cancer) was reported to have wild-type KRAS, PIK3CA, and PTEN, whereas P53 and BRAF are reported mutated [27]. Cells were kept at 37 °C and 5% CO_2_ in a humidified incubator. (The cell lines, HCT116 and HT29, were provided by Dr. Sudip Sen’s Lab, All India Institute of Medical Sciences, Delhi, as a kind gift).

### 4.3. Development of Regorafenib-Resistant HCT116 Cell Line

For developing the regorafenib-resistant cell line model HCT116, cells were treated with a low concentration (10 nM) of regorafenib and the drug concentration was gradually increased until the cells could tolerate the drug. The cells were monitored regularly for their tolerance to the drug and maintained in the presence of a low concentration of the drug. We, thus, labeled this cell line as REG-HCT116-R.

### 4.4. Cell Viability Assay and Combination Index

The cell viability of the cells against Ru-1 and regorafenib, both alone and in combinations, was assessed using the MTT assay. The MTT, a colorimetric assay for determining cell viability, was used to measure cytotoxicity. This assay is based on the cellular enzymes known as oxidoreductases, which are required for the reduction of the tetrazolium dye MTT to its insoluble, purple formazan crystals [47]. Hence, the higher the intensity of purple color formation, the greater the number of live cells. In 96-well cell culture plates (Thermo Cat No.167008), 1 × 10^4^ cells/well of HCT116, REG-R-HCT116, and HT29 were seeded and allowed to grow for 24 h. Cells were treated with Ru-1 and regorafenib of the concentration gradient from 0.125 to 16 µM for 48 h. Then, 50 µg/100 µL of MTT (G-Biosciences, Noida, India Cat No. #RC1130) was added to the media post treatment, and the cells were incubated in a CO_2_ incubator for 2–3 h. The foramzan crystals thus formed were resuspended in 100 µL DMSO (Fisher Scientific Prod. No. 12435). The absorbance of these cells was measured in an iMark Bio-Rad India microplate reader at 595nm. Experiments were repeated thrice. IC_50_ values were computed using the entire dose–response curve, with drug concentrations showing 50% inhibition of cell number compared with cells with no drug cultured simultaneously. By using Compusyn Software, the combination index (CI) and effects of both drugs according to the quantitative determination were computed (Biosoft, Inc., Ferguson, MO, USA). CI > 1 denotes a synergistic impact, CI = 1 denotes an additive effect, and CI < 1 denotes antagonism [48].

### 4.5. Propidium Iodide Staining (PI)

To confirm the MTT assay results, PI live–dead exclusion assay is performed. PI is a membrane-impermeable dye, and the plasma membrane of live cells does not allow the dye to enter inside the cells. In late apoptotic and necrotic cells, plasma and nuclear membranes lose their integrity, allowing PI to pass through them, intercalate the DNA, and display red fluorescence [49]. All three cell lines were seeded into a 6-well culture plate with 5 × 10^4^ cells per well, and the cells were then cultured in a CO_2_ incubator for 24 h. The cells were treated with different concentrations of Ru-1 and regorafenib, alone and in combination, for 48 h. Cells were stained with 10 µg/mL PI following treatment. (PI, HIMEDIA, and REF ML067) and imaged using a Leica fluorescence microscope in red emission wavelength of monochromatic light at 10× magnification.

### 4.6. Colony Formation Assay

Cells were seeded onto a cell-culture-grade 12-well plate (from Greiner bio one), where 500 cells per well were seeded from both the cell lines, HCT116 and REG-HCT116-R, and cultured overnight at 37 °C with 5% CO_2_. The next day, cells were treated with different concentrations of regorafenib or Ru-1 or both for approximately two weeks until colonies were significantly visible; culture media with no drug was used as control. Fresh drug-treated media was replaced every second day during this process. At the termination of the experiment, colonies were fixed with 100% methanol for 20 min at room temperature after two PBS washes. After that, they were washed with water and the colonies stained with 0.5% crystal violet for 5 min, extra stain was washed off, and the plate was inverted over tissue paper overnight for drying. The number of colonies was counted and imaged under the microscope.

### 4.7. Mitochondrial Membrane Potential Loss JC-1 Staining

To determine whether cell death occurred due to apoptosis, we looked for a hallmark of cell death due to apoptosis and membrane potential loss (MMP). MMP was evaluated by staining the drug-treated cells with JC-1 dye. The lipophilic, cationic JC-1 dye can enter mitochondria, where it accumulates and forms reversible complexes known as J-aggregates, which exhibit red fluorescence. The dye naturally exhibits green fluorescence [50]. Hence, the JC-1 dye enters and builds up in the energised mitochondria, carrying a negative charge in healthy cells and naturally forms red fluorescent J-aggregates. In unhealthy or apoptotic cells, the membrane becomes more permeable and loses its integrity, allowing JC-1 dye to leak from the mitochondria and exists as J-monomers, exhibiting green fluorescence. Six-well plates were seeded with 5 × 10^5^ cells per well from all three cell lines, and the plates were incubated for 24 h to allow the cells to adhere to the surface, after which they were treated with varying concentrations of the two drugs and their combinations, as defined before. Post 24 h of treatment, JC-1 dye was added to each well with a working concentration of 2 µM for 20 min. Then, the cells were examined by fluorescence microscopy with Leica Microscope 20× magnification using standard filters for TRITC and FITC. Additionally, the stained cells were used for flow cytometric analysis using a Beckman Coulter Cytoflex flow cytometer, and CytExpert software was used to evaluate the data. To verify the loss of MMP during apoptosis, 50 mM Carbonyl cyanide m-chlorophenyl hydrazine (CCCP, G-BIOSCIENCES, Noida, India) was taken as a positive control [51].

### 4.8. Reactive Oxygen Species (ROS) Study

Using a Leica-fluorescence microscope with an emission wavelength of 530 nm, the dichlorofluorescein (DCFH) assay was used to quantify the cellular oxidative stress caused in the Ru-1, REG, and REG/Ru-1 drug response. DCFH-DA a fluorescent probe is utilised to find intracellular oxidative stress. In the presence of ROS, DCFH-DA is hydrolysed to DCFH and kept intracellularly. Fluorescent dichlorofluorescein, which fluoresces green, is produced when DCFH is oxidised [24]. Cells are plated in 6-well plates at a density of 2.5 × 10^5^ cells and allowed to grow in a CO_2_ incubator for 24 h. Cells were then treated with regorafenib and Ru-1 alone and in different combinations for 48 h. After that, cells were rinsed with 1X PBSand, stained with 5 µM DFCH-DA (G-Biosciences Cat. #RC1066), and incubated at 37 °C for 20 min. A Leica fluorescence microscope was used to image the cells using a FITC filter; a 20× magnification was used for HT29 cells, and 10× magnification was used for imaging HCT116 and REG-HCT116-R [52].

### 4.9. Scratch and Wound Healing Assay

In a 12-well plate, 1 × 10^4^ cells per well for each of the three cell lines were seeded with 60% confluency. In advance, a horizontal line running through the center of the bottom of each well was used to mark it. Using a 10 µL tip, the scratch was made in the center of each well. Treatment was given later with varying concentrations of the two drugs, regorafenib and Ru-1, and their combinations, as defined before. The cells were imaged after 0, 24, and 48 h of treatment, and changes in the scratch were examined. The microscopy was conducted with a Leica microscope using a standard DIC filter at 10× magnification [53].

### 4.10. Real-Time-qPCR

Cells treated with regorafenib and Ru-1 at IC-50 concentrations, alone and in combinations, for 24 hrs, were collected by trypsinization, and using a QIAGEN Total RNA isolation kit (Germany Hilden #74134), total RNA was extracted. Nano-Drop (Colibri Titertek Berthold) was used to quantify total RNA. Using Thermo versco cDNA synthesis kit (Reg AB-1453/A), cDNA was synthesised using 1 µg of total RNA. Real-time-qPCR was performed with cDNA using SYBR Green Master Mix (Takara Cat No. RR420A, Shiga, Japan) in a Bio-Rad CFX Connect (Bio-Rad Laboratories, Hercules, CA, USA) with the following set of oligonucleotide primers: *PI3K*, 5′-GCTTGATGGATTTACTCTGG-3′, and 5′-GAGGTGCTCACAACTTCAAT-3′; *AKT*, 5′-AACTATCCTGCGGGTTTTA-3′, and 5′-CCTTAACATTTCCCTACGTG-3′; *Casp9*, 5′-GCTCTTCCTTTGTTCATCTC-3′, and 5′-GTAAGGTTTTCTAGGGTTGG-3′; *Bcl-2*, 5′-CGATCTGGAAATCCTCCTAA-3′, and 5′-CCATCAATCTTCAGCACTCT-3′; and *18S*, 5′-GCAATTATTCCCCATGAACG-3′, and 5′-GGCCTCACTAAACCATCCAA-3′.The conventional delta Ct method was used to calculate relative mRNA expression levels. As an internal control, 18S rRNA was used. [54,55].

### 4.11. Immunoblotting

The expression of different proteins during drug treatment was evaluated by extracting total proteins by protein lysis buffer (Promega #G938A, Madison, WI, USA) with protease (Roche #0589279100, Basel, Switzerland) and phosphatase inhibitors (Roche Phos STOP # 04906845001). Equivalent amounts of proteins were resolved on SDS PAGE. Proteins were transferred onto a PVDF membrane (MDI #SYFX8302XXXX101 Pore size 0.45µm) after being resolved. The membrane was then probed with the primary antibodies overnight at 4 degrees in the cold room with moderate shaking after being blocked with 5% non-fat skimmed milk (HI Media India #G938A) for non-phosphorylated proteins and with 5% BSA for phosphoproteins at room temperature for 1 h: Beta-actin antibody (1:5000) (ImmunoTag, Cat No. ITT07018, S. Rabbit, St. Louis, MO, USA), PI3K (1:1000) (BD-Bioscience Cat No. 611342 S. Mouse), Akt (1:1000) (BD-Bioscience Cat No. 610860 S. Mouse), p-Akt (Ser 473) (1:1000) (ImmunoTag, G-biosciences, Cat No. ITP0016, S. Rabbit), ERK (1:1000) (BD-Biosciences, Cat No. 610123, S. Mouse), p44/42-ERK1/2 (Thr202/Tyr204 and Thr185/Tyr187) (1:1000) (ImmunoTag, G-biosciences, Cat No. ITT00412 S. Mouse), P38 MAPK(pT180/Py182) (1:1000) (BD-Biosciences, Cat No. 612280, S. Mouse), anti-JNK/SAPK1 (1:1000) (BD-Biosciences, Cat No. 610627, S. Mouse), anti-VEGFA (1:1000) (Cloud-clone, Cat No. PAA143Hu01), anti-Bcl-2 (1:1000) (ImmunoTag, G-biosciences, Cat No. ITM3041, S. Mouse), and cleaved PARP (1:1000) (ImmunoTag, G-biosciences, Cat No. ITT07023, S. Rabbit). The blots were probed with respective secondary antibodies (Goat anti-Rabbit IgG/HRP 1:5000 ImmunoTag G-biosciences, Cat No. SE134 and Goat anti-Mouse 1:5000 BD Biosciences Cat No. 554002) and detection was carried out by the enhanced chemiluminescence (ECL) detection system.

### 4.12. Statistical Analysis

The mean and ± standard deviations were used to represent the data. A Student’s *t*-test with two-tailed analysis was used for comparison between control and treatments. Cell viability was tested for eight concentrations and three different drug treatments in triplicates. Since there were two variables, we performed two-way ANOVA to check whether the drug treatment or drug concentration or interactions between the drug treatment and the concentration significantly affected cell viability. Following ANOVA, post hoc analysis using Tukey’s test was performed for pairwise comparisons. A similar analysis was conducted separately for three cell lines. *p*-values of 0.05 or less were considered significant. Using CompuSyn software ver.1.0 (Combosyn, lnc., Paramus, NJ, 07652, USA), the combination index (CI) was determined using the Chou–Talalay approach to evaluate the synergistic efficacy of drug combinations.

## Figures and Tables

**Table 1:** IC_50_ values of Ru-1, regorafenib, and combination of Reg/Ru-1 in selected cancer cell lines.

Cell Line	Ru-1(µM)	REG (µM)	REG/Ru-1 (µM)
HCT116	0.9 ± 1.03	2.7 ± 0.6	0.4 ± 0.9
REG-HCT116-R	1.4 ± 1.0	9.9 ± 1.09	1.2 ± 0.96
HT29	0.6 ± 0.7	7.3 ± 0.98	0.9 ± 0.95

Data are expressed as Mean ± SD (n = 3). Statistical analysis was conducted using two-way ANOVA. Following ANOVA, post hoc analysis using Tukey’s test was performed for pairwise comparisons of the IC_50_ values of each cell line for each drug treatments, and *p*-values were found to be <0.0001.

**Table 2:** The synergistic effect of the ΔRu-1/Reg combination in selected cell lines—(1) HCT116, (2) regorafenib-resistant HCT116, and (3) HT29.

RU-1	REG	HCT116		REG-HCT116-R		HT29	
CONC µM	CONC µM	Fa	CI	Fa	CI	Fa	CI
0.125	0.125	0.39	0.2	0.17	0.4	0.25	0.9
0.25	0.25	0.56	0.26	0.35	0.3	0.43	0.5
0.5	0.5	0.7	0.26	0.6	0.27	0.54	0.6
1	1	0.74	0.43	0.64	0.46	0.58	1.1
2	2	0.77	0.7	0.66	0.86	0.65	1.4
4	4	0.9	0.52	0.7	1.4	0.8	1.0
8	8	0.95	0.49	0.85	1.2	0.9	0.7
16	16	0.99	0.05	0.99	0.1	0.99	0.06

Fa, fraction affected, corresponding to the fraction of cell viability (%); CI, combination index, where CI < 1 indicates synergism, CI > 1 indicates antagonism, and CI = 1 indicates additive effect.

## Data Availability

Data presented in this study are available on request.

## Supplementary Materials

The following supporting information can be downloaded at: https://www.mdpi.com/article/10.3390/ijms24010686/s1.

## References

[1] Biller L.H., Schrag D. (2021). Diagnosis and Treatment of Metastatic Colorectal Cancer: A Review. JAMA.

[2] Dagogo-Jack I., Shaw A.T. (2018). Tumour heterogeneity and resistance to cancer therapies. Nat. Rev. Clin. Oncol..

[3] Zhang Z., Zhou L., Xie N., Nice E.C., Zhang T., Cui Y., Huang C. (2020). Overcoming cancer therapeutic bottleneck by drug repurposing. Signal Transduct. Target. Ther..

[4] Delude C. (2012). Combinations go on trial. Cancer Discov..

[5] Rosenberg B., Van Camp L., Krigas T. (1965). Inhibition of Cell Division in Escherichia coli by Electrolysis Products from a Platinum Electrode. Nature.

[6] Wong E., Giandornenico C.M. (1999). Current Status of Platinum-Based Antitumor Drugs. Chem. Rev..

[7] Wang X., Wang X., Guo Z. (2015). Functionalization of Platinum Complexes for Biomedical Applications. Acc. Chem. Res..

[8] Bergamo A., Gaiddon C., Schellens J.H.M., Beijnen J.H., Sava G. (2012). Approaching tumour therapy beyond platinum drugs: Status of the art and perspectives of ruthenium drug candidates. J. Inorg. Biochem..

[9] Wang X., Wang X., Jin S., Muhammad N., Guo Z. (2019). Stimuli-Responsive Therapeutic Metallodrugs. Chem. Rev..

[10] Thota S., Rodrigues D.A., Crans D.C., Barreiro E.J. (2018). Ru(II) Compounds: Next-Generation Anticancer Metallotherapeutics?. J. Med. Chem..

[11] Zhao Z., Gao P., You Y., Chen T. (2018). Cancer-Targeting Functionalization of Selenium-Containing Ruthenium Conjugate with Tumor Microenvironment-Responsive Property to Enhance Theranostic Effects. Chem. Eur. J..

[12] Zeng L., Gupta P., Chen Y., Wang E., Ji L., Chao H., Chen Z.-S. (2017). The development of anticancer ruthenium(II) complexes: From single molecule compounds to nanomaterials. Chem. Soc. Rev..

[13] Chen T., Liu Y., Zheng W.J., Liu J., Wong Y.S. (2010). Ruthenium polypyridyl complexes that induce mitochondria-mediated apoptosis in cancer cells. Inorg. Chem..

[14] Deng Z., Gao P., Yu L., Ma B., You Y., Chan L., Mei C., Chen T. (2017). Ruthenium complexes with phenylterpyridine derivatives target cell membrane and trigger death receptors-mediated apoptosis in cancer cells. Biomaterials.

[15] Meng X., Leyva M.L., Jenny M., Gross I., Benosman S., Fricker B., Harlepp S., Hébraud P., Boos A., Wlosik P. (2009). A ruthenium-containing organometallic compound reduces tumor growth through induction of the endoplasmic reticulum stress gene CHOP. Cancer Res..

[16] Li G., Sasaki T., Asahina S., Roy M.C., Mochizuki T., Koizumi K. (2017). Patching of Lipid Rafts by Molecular Self-Assembled Nanofibrils Suppresses Cancer Cell Migration. Chem.

[17] Hu X., Lu Y., Shi X., Yao T., Dong C., Shi S. (2019). Integrating in situ formation of nanozymes with mesoporous polydopamine for combined chemo, photothermal and hypoxia-overcoming photodynamic therapy. Chem. Commun..

[18] Hu X., Lu Y., Dong C., Zhao W., Wu X., Zhou L., Chen L., Yao T., Shi S. (2020). A RuII Polypyridyl Alkyne Complex Based Metal–Organic Frameworks for Combined Photodynamic/Photothermal/Chemotherapy. Chem. Eur. J..

[19] Liu J., Chen Y., Li G., Zhang P., Jin C., Zeng L., Ji L., Chao H. (2015). Ruthenium(II) polypyridyl complexes as mitochondria-targeted two-photon photodynamic anticancer agents. Biomaterials.

[20] Chakrabortty S., Agrawalla B.K., Stumper A., Vegi N.M., Fischer S., Reichardt C., Kögler M., Dietzek B., Feuring-Buske M., Buske C. (2017). Mitochondria Targeted Protein-Ruthenium Photosensitizer for Efficient Photodynamic Applications. J. Am. Chem. Soc..

[21] Vyas K.M., Sharma D., Magani S.K.J., Mobin S.M., Mukhopadhyay S. (2021). In vitro evaluation of cytotoxicity and antimetastatic properties of novel arene ruthenium(II)-tetrazolato compounds on human cancer cell lines. Appl. Organomet. Chem..

[22] Wei N., Chu E., Wu S.Y., Wipf P., Schmitz J.C. (2015). The cytotoxic effects of regorafenib in combination with protein kinase D inhibition in human colorectal cancer cells. Oncotarget.

[23] Victorelli S., Passos J.F. (2019). Reactive Oxygen Species Detection in Senescent Cells. Methods Mol. Biol..

[24] Li P., Zhou L., Zhao T., Liu X., Zhang P., Liu Y., Zheng X., Li Q. (2017). Caspase-9: Structure, mechanisms and clinical application. Oncotarget.

[25] Subramonian D., Phanhthilath N., Rinehardt H., Flynn S., Huo Y., Zhang J., Messer K., Mo Q., Huang S., Lesperance J. (2020). Regorafenib is effective against neuroblastoma in vitro and in vivo and inhibits the RAS/MAPK, PI3K/Akt/mTOR and Fos/Jun pathways. Br. J. Cancer.

[26] Yang J., Nie J., Ma X., Wei Y., Peng Y., Wei X. (2019). Targeting PI3K in cancer: Mechanisms and advances in clinical trials. Mol. Cancer.

[27] Berg K.C.G., Eide P.W., Eilertsen I.A., Johannessen B., Bruun J., Danielsen S.A., Bjørnslett M., Meza-Zepeda L.A., Eknæs M., Lind G.E. (2017). Multi-omics of 34 colorectal cancer cell lines—A resource for biomedical studies. Mol. Cancer.

[28] Weng M.C., Li M.-H., Chung J.G., Liu Y.-C., Wu J.-Y., Hsu F.-T., Wang H.-E. (2019). Apoptosis induction and AKT/NF-κB inactivation are associated with regroafenib-inhibited tumor progression in non-small cell lung cancer in vitro and in vivo. Biomed. Pharmacother..

[29] Kumar A., Rajendran V., Sethumadhavan R., Purohit R. (2013). AKT kinase pathway: A leading target in cancer research. Sci. World J..

[30] Strumberg D., Schultheis B. (2012). Regorafenib for cancer. Expert Opin. Investig. Drugs.

[31] Huynh H., Ngo V.C., Koong H.N., Poon D., Choo S.P., Thng C.H., Chow P., Ong H.S., Chung A., Soo K.C. (2009). Sorafenib and rapamycin induce growth suppression in mouse models of hepatocellular carcinoma. J. Cell Mol. Med..

[32] Refolo M.G., Lippolis C., Carella N., Cavallini A., Messa C., D’Alessandro R. (2018). Chlorogenic Acid Improves the Regorafenib Effects in Human Hepatocellular Carcinoma Cells. Int. J. Mol. Sci..

[33] Han R., Li S. (2018). Regorafenib delays the proliferation of hepatocellular carcinoma by inducing autophagy. Pharmazie.

[34] Dhillon A.S., Hagan S., Rath O., Kolch W. (2007). MAP kinase signalling pathways in cancer. Oncogene.

[35] Wilhelm S.M., Dumas J., Adnane L., Lynch M., Carter C.A., Schütz G., Thierauch K.-H., Zopf D. (2011). Regorafenib (BAY 73-4506): A new oral multikinase inhibitor of angiogenic, stromal and oncogenic receptor tyrosine kinases with potent preclinical antitumor activity. Int. J. Cancer.

[36] Kapitza S., Jakupec M.A., Uhl M., Keppler B.K., Marian B. (2005). The heterocyclic ruthenium(III) complex KP1019 (FFC14A) causes DNA damage and oxidative stress in colorectal tumor cells. Cancer Lett..

[37] Thornton T.M., Rincon M. (2009). Non-classical p38 map kinase functions: Cell cycle checkpoints and survival. Int. J. Biol. Sci..

[38] Lin K., Rong Y., Chen D., Zhao Z., Bo H., Qiao A., Hao X., Wang J. (2020). Combination of Ruthenium Complex and Doxorubicin Synergistically Inhibits Cancer Cell Growth by Down-Regulating PI3K/AKT Signaling Pathway. Front. Oncol..

[39] Baliza I.R.S., Silva S.L.R., de Santos L.S., Neto J.H.A., Dias R.B., Sales C.B.S., Rocha C.A.G., Soares M.B.P., Batista A.A., Bezerra D.P. (2019). Ruthenium Complexes With Piplartine Cause Apoptosis Through MAPK Signaling by a p53-Dependent Pathway in Human Colon Carcinoma Cells and Inhibit Tumor Development in a Xenograft Model. Front. Oncol..

[40] Tan B.J., Chiu G.N.C. (2013). Role of oxidative stress, endoplasmic reticulum stress and ERK activation in triptolide-induced apoptosis. Int. J. Oncol..

[41] Yu C.C., Huang S.Y., Chang S.F., Liao K.F., Chiu S.C. (2020). The Synergistic Anti-Cancer Effects of NVP-BEZ235 and Regorafenib in Hepatocellular Carcinoma. Molecules.

[42] Lin K., Zhao Z.Z., Bo H.B., Hao X.J., Wang J.Q. (2018). Applications of ruthenium complex in tumor diagnosis and therapy. Front. Pharmacol..

[43] Tangchirakhaphan S., Innajak S., Nilwarangkoon S., Tanjapatkul N., Mahabusrakum W., Watanapokasin R. (2018). Mechanism of apoptosis induction associated with ERK1/2 upregulation via goniothalamin in melanoma cells. Exp. Ther. Med..

[44] McCubrey J.A., Steelman L.S., Chappell W.H., Abrams S.L., Wong E.W.T., Chang F., Lehmann B., Terrian D.M., Milella M., Tafuri A. (2007). Roles of the Raf/MEK/ERK pathway in cell growth, malignant transformation and drug resistance. Biochim. Biophys. Acta Mol. Cell Res..

[45] Owuor E.D., Kong A.N.T. (2002). Antioxidants and oxidants regulated signal transduction pathways. Biochem. Pharmacol..

[46] Heffeter P., Atil B., Kryeziu K., Groza D., Koellensperger G., Körner W., Jungwirth U., Mohr T., Keppler B.K., Berger W. (2013). The ruthenium compound KP1339 potentiates the anticancer activity of sorafenib in vitro and in vivo. Eur. J. Cancer..

[47] Van Meerloo J., Kaspers G.J.L., Cloos J. (2011). Cell sensitivity assays: The MTT assay. Methods Mol. Biol..

[48] Ashton J.C. (2015). Drug combination studies and their synergy quantification using the Chou-Talalay method—Letter. Cancer Res..

[49] Riccardi C., Nicoletti I. (2006). Analysis of apoptosis by propidium iodide staining and flow cytometry. Nat. Protoc..

[50] Rasool F., Sharma D., Anand P.S., Magani S.K.J., Tantravahi S. (2021). Evaluation of the Anticancer Properties of Geranyl Isovalerate, an Active Ingredient of Argyreia nervosa Extract in Colorectal Cancer Cells. Front. Pharmacol..

[51] Sivandzade F., Bhalerao A., Cucullo L. (2019). Analysis of the Mitochondrial Membrane Potential Using the Cationic JC-1 Dye as a Sensitive Fluorescent Probe. Bio-Protocols.

[52] Wu D., Yotnda P. (2011). Production and Detection of Reactive Oxygen Species (ROS) in Cancers. J. Vis. Exp..

[53] Pijuan J., Barceló C., Moreno D.F., Maiques O., Sisó P., Marti M.R., Macià A., Panosa A. (2019). In vitro cell migration, invasion, and adhesion assays: From cell imaging to data analysis. Front. Cell Dev. Biol..

[54] Jozefczuk J., Adjaye J. (2011). Quantitative Real-Time PCR-Based Analysis of Gene Expression. Methods Enzymol..

[55] Ranjan A., Sharma D., Srivastava A.K., Varma A., Jayadev M.S.K., Joshi R.K. (2022). Evaluation of anticancer activity of ferrocene based benzothiazole and β-ketooxothioacetal. J. Organomet. Chem..

