# Supplementary material for: Regorafenib and Ruthenium Complex Combination Inhibit Cancer Cell Growth by Targeting PI3K/AKT/ERK Signalling in Colorectal Cancer Cells
Source: Int J Mol Sci. 2022 Dec 30;24(1):686. doi: 10.3390/ijms24010686 (PMC9820863; doi:10.3390/ijms24010686)
Supplement: Supplementary file 1 [file ijms-24-00686-s001.zip › Figures Supplementary.pdf]

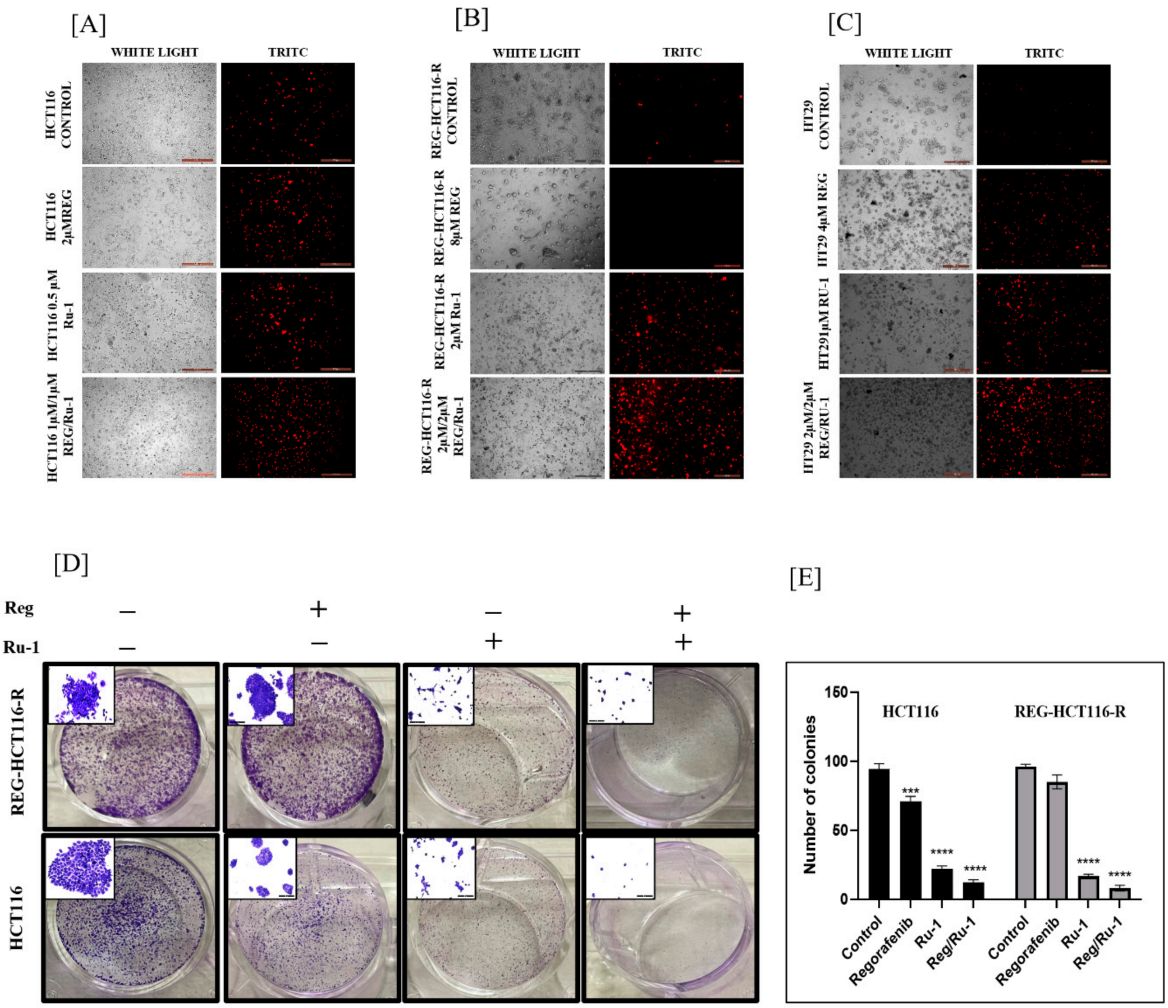

Figure S1. Propidium iodide staining for live/ dead cell detection

[A] HCT116 cells were treated with IC_{50} concentration of Reg alone, Ru-1 alone or in combination for 48 h showed increased positivity for redfluorescence when compared to that of cells treated with individual drugs and even control untreated cells.

[B] REG-HCT116-R cells were treated with IC_{50} concentration of Reg alone, Ru-1 alone or in combination for 48 h and showed increased positivity for red fluorescence when compared to cells treated with individual drugs and even control untreated cells.

[C] HT29 cells treatedwith Reg alone, Ru-1 alone or in combination for 48 h showed increased positivity for red fluorescence compared to cells treated with individual drugs and even control untreated cells. Scale bar 100 μm.

[D] Colony formation assay cells HCT116 and REG-HCT116-R cells treated with Reg alone, Ru-1 alone or in combination. (20X Magnification) Scale bar 200 μm.

[E] Bar graph represents number of colonies formed after treatment in HCT116 and REG-HCT116-R cells. *p < 0.05, **p < 0.01, ***p < 0.001.

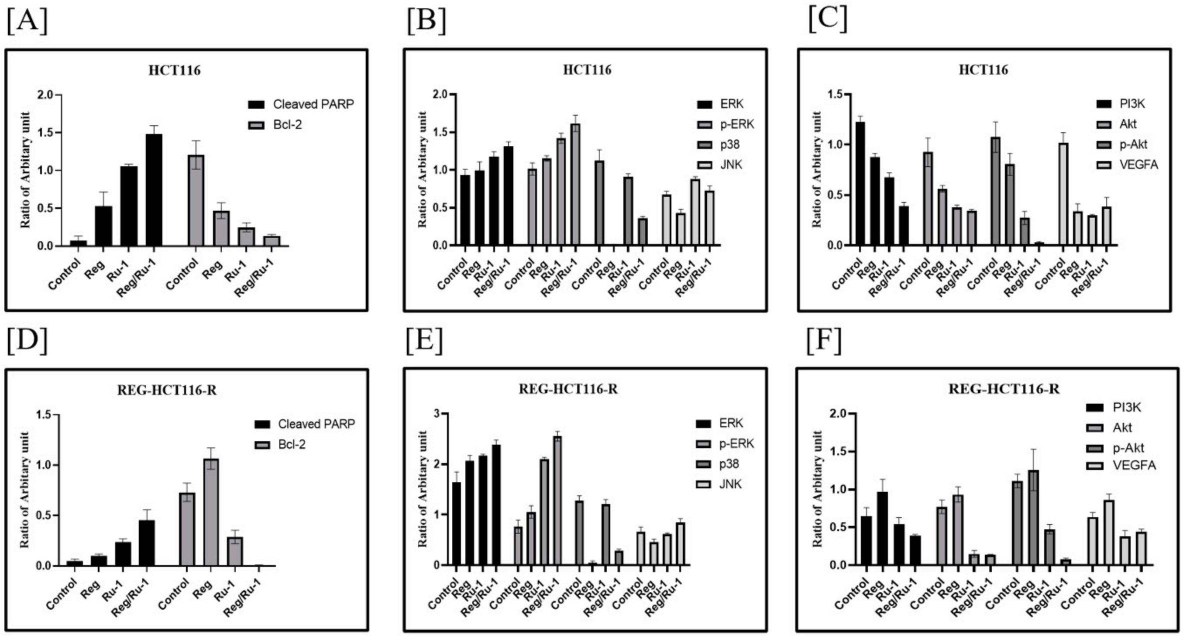

Figure S2. **[A] [B] [C]** Densitometric analysis of HCT116 cells western blots.
[D] [E] [F] Densitometric analysis of REG-HCT116-R cells western blots.
