# Supplementary material for: Regorafenib and Ruthenium Complex Combination Inhibit Cancer Cell Growth by Targeting PI3K/AKT/ERK Signalling in Colorectal Cancer Cells
Source: Int J Mol Sci. 2022 Dec 30;24(1):686. doi: 10.3390/ijms24010686 (PMC9820863; doi:10.3390/ijms24010686)
Supplement: Supplementary file 1 [file ijms-24-00686-s001.zip › Structures of Regorafenib and Ruthenium Supplementary.pdf]

Structures of Regorafenib and Ruthenium-1

REGORAFENIB

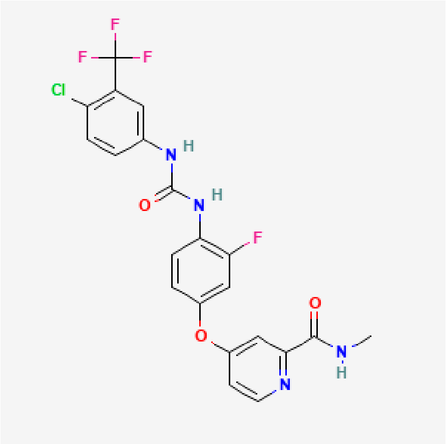

Reference-National Center for Biotechnology Information (2022). PubChem Compound Summary for CID 11167602, Regorafenib. Retrieved December 13, 2022, from https://pubchem.ncbi.nlm.nih.gov/compound/Regorafenib.

Ruthenium-1

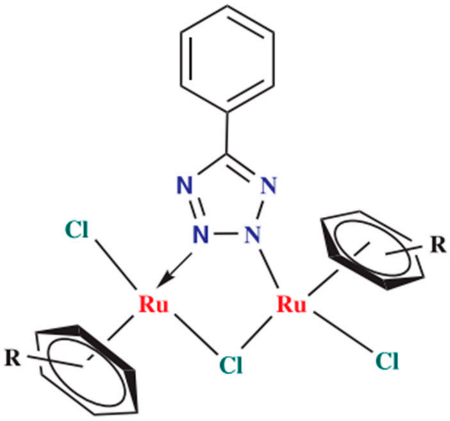

[Ru]-1

Reference-Vyas KM, Sharma D, Magani SKJ, Mobin SM, Mukhopadhyay S. In vitro evaluation of cytotoxicity and antimetastatic properties of novel arene ruthenium(II)-tetrazolato compounds on human cancer cell lines. Appl Organomet Chem. 2021;35(5):e6187. doi:10.1002/AOC.6187
