# Supplementary material for: Regorafenib and Ruthenium Complex Combination Inhibit Cancer Cell Growth by Targeting PI3K/AKT/ERK Signalling in Colorectal Cancer Cells
Source: Int J Mol Sci. 2022 Dec 30;24(1):686. doi: 10.3390/ijms24010686 (PMC9820863; doi:10.3390/ijms24010686)
Supplement: Supplementary file 1 [file ijms-24-00686-s001.zip › TWO-WAY ANOVA ANALYSIS SUPPLEMENTARY DATA.pdf]

FIXED-EFFECTS TWO-WAY ANOVA	Cell line HCT116				
	Sum of sqrs	df	Mean square	F	p (same)
Drug:	10147	2	5073.52	921.5	1.85E-42
Concentration:	64604	8	8075.5	1467	2.81E-60
Interaction:	3446.99	16	215.437	39.13	6.09E-24
Within:	297.311	54	5.50576		
Total:	78495.4	80			

Post hoc analysis of drug treatments	RU-1	REG	COMB
RU-1		23.64	36.61
REG	<0.0001		60.25
COMB	<0.0001	<0.0001	

Post hoc analysis of drug concentrations HCT116

	0	0.125	0.25	0.5	1	2	4	8	16
0		25.17	39.6	57.36	70.77	79.04	87.67	106.8	117.3
0.125	<0.0001		14.44	32.19	45.6	53.87	62.5	81.6	92.09
0.25	<0.0001	<0.0001		17.75	31.16	39.43	48.06	67.16	77.65
0.5	<0.0001	<0.0001	<0.0001		13.41	21.68	30.31	49.41	59.9
1	<0.0001	<0.0001	<0.0001	<0.0001		8.266	16.9	36	46.49
2	<0.0001	<0.0001	<0.0001	<0.0001	<0.0001		8.631	27.73	38.22
4	<0.0001	<0.0001	<0.0001	<0.0001	<0.0001	<0.0001		19.1	29.59
8	<0.0001	<0.0001	<0.0001	<0.0001	<0.0001	<0.0001	<0.0001		10.49
16	<0.0001	<0.0001	<0.0001	<0.0001	<0.0001	<0.0001	<0.0001	<0.0001	

Post hoc analysis of drug interactions HCT116

Drug & concentration	Drug & concentration	Tukey's (Q)	p
RU-1-0	RU-1-0.125	6.24	0.01127
RU-1-0	RU-1-0.25	11.73	1.11E-08
RU-1-0	RU-1-0.5	33.9	4.37E-13
RU-1-0	RU-1-1	42.61	4.37E-13
RU-1-0	RU-1-2	46.13	4.37E-13
RU-1-0	RU-1-4	48.37	4.37E-13
RU-1-0	RU-1-8	64.05	4.37E-13
RU-1-0	RU-1-16	70.96	4.37E-13
RU-1-0	REG-0	0	1

RU-1-0	COMB-0	0	1
RU-1-0.125	RU-1-0.25	5.493	0.05181
RU-1-0.125	RU-1-0.5	27.66	4.37E-13
RU-1-0.125	RU-1-1	36.37	4.37E-13
RU-1-0.125	RU-1-2	39.89	4.37E-13
RU-1-0.125	RU-1-4	42.13	4.37E-13
RU-1-0.125	RU-1-8	57.81	4.37E-13
RU-1-0.125	RU-1-16	64.72	4.37E-13
RU-1-0.125	REG-0.125	3.602	0.6896
RU-1-0.125	COMB-0.125	21.27	4.38E-13
RU-1-0.25	RU-1-0.5	22.17	4.38E-13
RU-1-0.25	RU-1-1	30.87	4.37E-13
RU-1-0.25	RU-1-2	34.4	4.37E-13
RU-1-0.25	RU-1-4	36.64	4.37E-13
RU-1-0.25	RU-1-8	52.31	4.37E-13
RU-1-0.25	RU-1-16	59.23	4.37E-13
RU-1-0.25	REG-0.25	4.26	0.3664
RU-1-0.25	COMB-0.25	29.14	4.37E-13
RU-1-0.5	RU-1-1	8.705	2.96E-05
RU-1-0.5	RU-1-2	12.23	3.06E-09
RU-1-0.5	RU-1-4	14.47	1.11E-11
RU-1-0.5	RU-1-8	30.14	4.37E-13
RU-1-0.5	RU-1-16	37.06	4.37E-13
RU-1-0.5	REG-0.5	16.38	6.31E-13
RU-1-0.5	COMB-0.5	14.03	3.19E-11
RU-1-1	RU-1-2	3.523	0.7272
RU-1-1	RU-1-4	5.763	0.03054
RU-1-1	RU-1-8	21.44	4.38E-13
RU-1-1	RU-1-16	28.36	4.37E-13
RU-1-1	REG-1	16.6	5.72E-13
RU-1-1	COMB-1	11.35	2.98E-08
RU-1-2	RU-1-4	2.24	0.9972
RU-1-2	RU-1-8	17.92	4.69E-13
RU-1-2	RU-1-16	24.83	4.37E-13
RU-1-2	REG-2	12.57	1.26E-09
RU-1-2	COMB-2	11.08	6.15E-08
RU-1-4	RU-1-8	15.68	1.11E-12
RU-1-4	RU-1-16	22.59	4.38E-13
RU-1-4	REG-4	8.188	0.00011
RU-1-4	COMB-4	14.92	4.03E-12
RU-1-8	RU-1-16	6.916	0.002456
RU-1-8	REG-8	12.76	7.73E-10
RU-1-8	COMB-8	5.553	0.04621
RU-1-16	REG-16	12.28	2.69E-09
RU-1-16	COMB-16	2.485	0.9889
REG-0	REG-0.125	9.842	1.56E-06

REG-0	REG-0.25	15.99	8.08E-13
REG-0	REG-0.5	17.52	4.82E-13
REG-0	REG-1	26.01	4.37E-13
REG-0	REG-2	33.56	4.37E-13
REG-0	REG-4	40.18	4.37E-13
REG-0	REG-8	51.28	4.37E-13
REG-0	REG-16	58.68	4.37E-13
REG-0	COMB-0	0	1
REG-0.125	REG-0.25	6.151	0.01364
REG-0.125	REG-0.5	7.677	0.000395
REG-0.125	REG-1	16.17	7.16E-13
REG-0.125	REG-2	23.71	4.37E-13
REG-0.125	REG-4	30.34	4.37E-13
REG-0.125	REG-8	41.44	4.37E-13
REG-0.125	REG-16	48.84	4.37E-13
REG-0.125	COMB-0.125	17.67	4.74E-13
REG-0.25	REG-0.5	1.526	1
REG-0.25	REG-1	10.02	9.88E-07
REG-0.25	REG-2	17.56	4.80E-13
REG-0.25	REG-4	24.19	4.37E-13
REG-0.25	REG-8	35.29	4.37E-13
REG-0.25	REG-16	42.69	4.37E-13
REG-0.25	COMB-0.25	24.88	4.37E-13
REG-0.5	REG-1	8.49	5.14E-05
REG-0.5	REG-2	16.04	7.88E-13
REG-0.5	REG-4	22.66	4.38E-13
REG-0.5	REG-8	33.76	4.37E-13
REG-0.5	REG-16	41.16	4.37E-13
REG-0.5	COMB-0.5	30.41	4.37E-13
REG-1	REG-2	7.548	0.000542
REG-1	REG-4	14.17	2.25E-11
REG-1	REG-8	25.27	4.37E-13
REG-1	REG-16	32.67	4.37E-13
REG-1	COMB-1	27.95	4.37E-13
REG-2	REG-4	6.624	0.004805
REG-2	REG-8	17.73	4.72E-13
REG-2	REG-16	25.13	4.37E-13
REG-2	COMB-2	23.65	4.37E-13
REG-4	REG-8	11.1	5.76E-08
REG-4	REG-16	18.5	4.53E-13
REG-4	COMB-4	23.11	4.38E-13
REG-8	REG-16	7.4	0.000776
REG-8	COMB-8	18.32	4.57E-13
REG-16	COMB-16	14.76	5.66E-12
COMB-0	COMB-0.125	27.51	4.37E-13
COMB-0	COMB-0.25	40.87	4.37E-13
COMB-0	COMB-0.5	47.93	4.37E-13
COMB-0	COMB-1	53.96	4.37E-13

COMB-0	COMB-2	57.21	4.37E-13
COMB-0	COMB-4	63.29	4.37E-13
COMB-0	COMB-8	69.6	4.37E-13
COMB-0	COMB-16	73.45	4.37E-13
COMB-0.125	COMB-0.25	13.36	1.68E-10
COMB-0.125	COMB-0.5	20.42	4.39E-13
COMB-0.125	COMB-1	26.45	4.37E-13
COMB-0.125	COMB-2	29.7	4.37E-13
COMB-0.125	COMB-4	35.78	4.37E-13
COMB-0.125	COMB-8	42.09	4.37E-13
COMB-0.125	COMB-16	45.94	4.37E-13
COMB-0.25	COMB-0.5	7.059	0.001757
COMB-0.25	COMB-1	13.09	3.36E-10
COMB-0.25	COMB-2	16.34	6.44E-13
COMB-0.25	COMB-4	22.42	4.38E-13
COMB-0.25	COMB-8	28.73	4.37E-13
COMB-0.25	COMB-16	32.58	4.37E-13
COMB-0.5	COMB-1	6.031	0.01761
COMB-0.5	COMB-2	9.276	6.79E-06
COMB-0.5	COMB-4	15.36	1.77E-12
COMB-0.5	COMB-8	21.67	4.38E-13
COMB-0.5	COMB-16	25.52	4.37E-13
COMB-1	COMB-2	3.245	0.8432
COMB-1	COMB-4	9.33	5.90E-06
COMB-1	COMB-8	15.64	1.17E-12
COMB-1	COMB-16	19.49	4.44E-13
COMB-2	COMB-4	6.085	0.01571
COMB-2	COMB-8	12.39	2.01E-09
COMB-2	COMB-16	16.24	6.78E-13
COMB-4	COMB-8	6.308	0.009725
COMB-4	COMB-16	10.16	6.84E-07
COMB-8	COMB-16	3.848	0.5663

FIXED-EFFECTS TWO-WAY ANOVA	REG-HCT116-R				
	Sum of sqrs	df	Mean square	F	p (same)
Drug:	20385.9	2	10192.9	4867	1.06E-61
Concentration:	55307.5	8	6913.44	3301	9.33E-70
Interaction:	5329.66	16	333.103	159	1.76E-39
Within:	113.096	54	2.09437		
Total:	81136.2	80			

Post hoc analysis of drug treatments and drug concentrations REG-HCT116-R

Drug	RU-1	REG	COMB
RU-1		99.29	35.25

REG	<0.0001		134.5
COMB	<0.0001	<0.0001	

concentrations	0	0.125	0.25	0.5	1	2	4	8	16
0		14.43	33.63	74.5	89.42	96.7	107.3	143.4	171.7
0.125	<0.0001		19.19	60.06	74.98	82.26	92.9	128.9	157.3
0.25	<0.0001	<0.0001		40.87	55.79	63.07	73.71	109.7	138.1
0.5	<0.0001	<0.0001	<0.0001		14.92	22.2	32.84	68.86	97.23
1	<0.0001	<0.0001	<0.0001	<0.0001		7.28	17.92	53.94	82.31
2	<0.0001	<0.0001	<0.0001	<0.0001	<0.0001		10.64	46.66	75.02
4	<0.0001	<0.0001	<0.0001	<0.0001	<0.0001	<0.0001		36.02	64.39
8	<0.0001	<0.0001	<0.0001	<0.0001	<0.0001	<0.0001	<0.0001		28.37
16	<0.0001	<0.0001	<0.0001	<0.0001	<0.0001	<0.0001	<0.0001	<0.0001	

Post hoc analysis of drug concentrations REG-HCT116-R

Drug & concentration	Drug & concentration	Tukey's Q	p
RU-1-0	RU-1-0.125	4.049	0.4654
RU-1-0	RU-1-0.25	15.96	8.28E-13
RU-1-0	RU-1-0.5	53.06	4.37E-13
RU-1-0	RU-1-1	63.43	4.37E-13
RU-1-0	RU-1-2	70.04	4.37E-13
RU-1-0	RU-1-4	73.84	4.37E-13
RU-1-0	RU-1-8	95.26	4.37E-13
RU-1-0	RU-1-16	110.5	4.37E-13
RU-1-0	REG-0	0	1
RU-1-0	COMB-0	0	1
RU-1-0.125	RU-1-0.25	11.91	7.02E-09
RU-1-0.125	RU-1-0.5	49.01	4.37E-13
RU-1-0.125	RU-1-1	59.38	4.37E-13
RU-1-0.125	RU-1-2	65.99	4.37E-13
RU-1-0.125	RU-1-4	69.79	4.37E-13
RU-1-0.125	RU-1-8	91.21	4.37E-13
RU-1-0.125	RU-1-16	106.5	4.37E-13
RU-1-0.125	REG-0.125	4.049	0.4654
RU-1-0.125	COMB-0.125	16.9	5.26E-13
RU-1-0.25	RU-1-0.5	37.1	4.37E-13
RU-1-0.25	RU-1-1	47.47	4.37E-13
RU-1-0.25	RU-1-2	54.08	4.37E-13
RU-1-0.25	RU-1-4	57.88	4.37E-13
RU-1-0.25	RU-1-8	79.3	4.37E-13
RU-1-0.25	RU-1-16	94.55	4.37E-13
RU-1-0.25	REG-0.25	15.96	8.28E-13
RU-1-0.25	COMB-0.25	26.33	4.37E-13
RU-1-0.5	RU-1-1	10.37	3.88E-07
RU-1-0.5	RU-1-2	16.98	5.18E-13

RU-1-0.5	RU-1-4	20.78	4.39E-13
RU-1-0.5	RU-1-8	42.2	4.37E-13
RU-1-0.5	RU-1-16	57.44	4.37E-13
RU-1-0.5	REG-0.5	49.55	4.37E-13
RU-1-0.5	COMB-0.5	19.4	4.46E-13
RU-1-1	RU-1-2	6.605	0.005014
RU-1-1	RU-1-4	10.41	3.54E-07
RU-1-1	RU-1-8	31.83	4.37E-13
RU-1-1	RU-1-16	47.07	4.37E-13
RU-1-1	REG-1	49	4.37E-13
RU-1-1	COMB-1	13.58	9.78E-11
RU-1-2	RU-1-4	3.803	0.5895
RU-1-2	RU-1-8	25.22	4.37E-13
RU-1-2	RU-1-16	40.47	4.37E-13
RU-1-2	REG-2	50.57	4.37E-13
RU-1-2	COMB-2	7.938	0.000207
RU-1-4	RU-1-8	21.42	4.38E-13
RU-1-4	RU-1-16	36.66	4.37E-13
RU-1-4	REG-4	43.89	4.37E-13
RU-1-4	COMB-4	8.281	8.73E-05
RU-1-8	RU-1-16	15.24	2.17E-12
RU-1-8	REG-8	42.97	4.37E-13
RU-1-8	COMB-8	5.49	0.05214
RU-1-16	REG-16	41.89	4.37E-13
RU-1-16	COMB-16	7.815	0.000281
REG-0	REG-0.125	0	1
REG-0	REG-0.25	0	1
REG-0	REG-0.5	3.511	0.7329
REG-0	REG-1	14.43	1.20E-11
REG-0	REG-2	19.47	4.44E-13
REG-0	REG-4	29.95	4.37E-13
REG-0	REG-8	52.29	4.37E-13
REG-0	REG-16	68.61	4.37E-13
REG-0	COMB-0	0	1
REG-0.125	REG-0.25	0	1
REG-0.125	REG-0.5	3.511	0.7329
REG-0.125	REG-1	14.43	1.20E-11
REG-0.125	REG-2	19.47	4.44E-13
REG-0.125	REG-4	29.95	4.37E-13
REG-0.125	REG-8	52.29	4.37E-13
REG-0.125	REG-16	68.61	4.37E-13
REG-0.125	COMB-0.125	20.95	4.39E-13
REG-0.25	REG-0.5	3.511	0.7329
REG-0.25	REG-1	14.43	1.20E-11
REG-0.25	REG-2	19.47	4.44E-13
REG-0.25	REG-4	29.95	4.37E-13

REG-0.25	REG-8	52.29	4.37E-13
REG-0.25	REG-16	68.61	4.37E-13
REG-0.25	COMB-0.25	42.29	4.37E-13
REG-0.5	REG-1	10.92	9.17E-08
REG-0.5	REG-2	15.96	8.24E-13
REG-0.5	REG-4	26.44	4.37E-13
REG-0.5	REG-8	48.78	4.37E-13
REG-0.5	REG-16	65.1	4.37E-13
REG-0.5	COMB-0.5	68.95	4.37E-13
REG-1	REG-2	5.038	0.1175
REG-1	REG-4	15.52	1.39E-12
REG-1	REG-8	37.86	4.37E-13
REG-1	REG-16	54.18	4.37E-13
REG-1	COMB-1	62.58	4.37E-13
REG-2	REG-4	10.48	2.94E-07
REG-2	REG-8	32.82	4.37E-13
REG-2	REG-16	49.14	4.37E-13
REG-2	COMB-2	58.5	4.37E-13
REG-4	REG-8	22.34	4.38E-13
REG-4	REG-16	38.66	4.37E-13
REG-4	COMB-4	52.17	4.37E-13
REG-8	REG-16	16.32	6.46E-13
REG-8	COMB-8	48.46	4.37E-13
REG-16	COMB-16	49.71	4.37E-13
COMB-0	COMB-0.125	20.95	4.39E-13
COMB-0	COMB-0.25	42.29	4.37E-13
COMB-0	COMB-0.5	72.46	4.37E-13
COMB-0	COMB-1	77.01	4.37E-13
COMB-0	COMB-2	77.98	4.37E-13
COMB-0	COMB-4	82.12	4.37E-13
COMB-0	COMB-8	100.7	4.37E-13
COMB-0	COMB-16	118.3	4.37E-13
COMB-0.125	COMB-0.25	21.34	4.38E-13
COMB-0.125	COMB-0.5	51.51	4.37E-13
COMB-0.125	COMB-1	56.06	4.37E-13
COMB-0.125	COMB-2	57.03	4.37E-13
COMB-0.125	COMB-4	61.17	4.37E-13
COMB-0.125	COMB-8	79.8	4.37E-13
COMB-0.125	COMB-16	97.37	4.37E-13
COMB-0.25	COMB-0.5	30.18	4.37E-13
COMB-0.25	COMB-1	34.72	4.37E-13
COMB-0.25	COMB-2	35.69	4.37E-13
COMB-0.25	COMB-4	39.83	4.37E-13
COMB-0.25	COMB-8	58.46	4.37E-13
COMB-0.25	COMB-16	76.03	4.37E-13
COMB-0.5	COMB-1	4.546	0.2521

COMB-0.5	COMB-2	5.513	0.04992
COMB-0.5	COMB-4	9.658	2.51E-06
COMB-0.5	COMB-8	28.29	4.37E-13
COMB-0.5	COMB-16	45.86	4.37E-13
COMB-1	COMB-2	0.9664	1
COMB-1	COMB-4	5.112	0.1036
COMB-1	COMB-8	23.74	4.37E-13
COMB-1	COMB-16	41.31	4.37E-13
COMB-2	COMB-4	4.146	0.419
COMB-2	COMB-8	22.77	4.38E-13
COMB-2	COMB-16	40.34	4.37E-13
COMB-4	COMB-8	18.63	4.51E-13
COMB-4	COMB-16	36.2	4.37E-13
COMB-8	COMB-16	17.57	4.79E-13

FIXED-EFFECTS TWO-WAY ANOVA			HT-29		
	Sum of sqrs	df	Mean square	F	p (same)
Drug:	19138.1	2	9569.06	2213	1.56E-52
Concentration:	49443.8	8	6180.48	1429	5.65E-60
Interaction:	4542.42	16	283.901	65.65	1.57E-29
Within:	233.537	54	4.32475		
Total:	73357.9	80			

Post hoc analysis of drug concentrations HT29

Drug	RU-1	REG	COMB
RU-1		74.29	12.85
REG	<0.0001		87.13
COMB	<0.0001	<0.0001	

Post hoc analysis of drug concentrations HT29

concentrations	0	0.125	0.25	0.5	1	2	4	8	16
0		32.21	44.66	52.19	56.14	63.5	81.54	107.2	124
0.125	<0.0001		12.46	19.99	23.94	31.29	49.33	75.04	91.76
0.25	<0.0001	<0.0001		7.53	11.48	18.84	36.87	62.59	79.31
0.5	<0.0001	<0.0001	<0.0001		3.953	11.31	29.34	55.06	71.78
1	<0.0001	<0.0001	<0.0001	0.1408		7.354	25.39	51.1	67.82
2	<0.0001	<0.0001	<0.0001	<0.0001	<0.0001		18.04	43.75	60.47
4	<0.0001	<0.0001	<0.0001	<0.0001	<0.0001	<0.0001		25.71	42.43
8	<0.0001	<0.0001	<0.0001	<0.0001	<0.0001	<0.0001	<0.0001		16.72
16	<0.0001	<0.0001	<0.0001	<0.0001	<0.0001	<0.0001	<0.0001	<0.0001	

Post hoc analysis of drug interactions HT29

Drug & concentration	Drug & concentration	Tukey's Q	p
RU-1-0	RU-1-0.125	35.52	4.37E-13
RU-1-0	RU-1-0.25	37.06	4.37E-13
RU-1-0	RU-1-0.5	37.69	4.37E-13
RU-1-0	RU-1-1	41.13	4.37E-13
RU-1-0	RU-1-2	37.37	4.37E-13
RU-1-0	RU-1-4	45.28	4.37E-13
RU-1-0	RU-1-8	71.14	4.37E-13
RU-1-0	RU-1-16	80.39	4.37E-13
RU-1-0	REG-0	0	1
RU-1-0	COMB-0	0	1
RU-1-0.125	RU-1-0.25	1.54	1
RU-1-0.125	RU-1-0.5	2.172	0.9982
RU-1-0.125	RU-1-1	5.612	0.0412
RU-1-0.125	RU-1-2	1.844	0.9999
RU-1-0.125	RU-1-4	9.754	1.96E-06
RU-1-0.125	RU-1-8	35.62	4.37E-13
RU-1-0.125	RU-1-16	44.87	4.37E-13
RU-1-0.125	REG-0.125	35.24	4.37E-13
RU-1-0.125	COMB-0.125	15.54	1.34E-12
RU-1-0.25	RU-1-0.5	0.6318	1
RU-1-0.25	RU-1-1	4.072	0.4544
RU-1-0.25	RU-1-2	0.3039	1
RU-1-0.25	RU-1-4	8.214	0.0001035
RU-1-0.25	RU-1-8	34.08	4.37E-13
RU-1-0.25	RU-1-16	43.33	4.37E-13
RU-1-0.25	REG-0.25	35.12	4.37E-13
RU-1-0.25	COMB-0.25	1.287	1
RU-1-0.5	RU-1-1	3.44	0.765
RU-1-0.5	RU-1-2	0.3279	1
RU-1-0.5	RU-1-4	7.582	0.0004984
RU-1-0.5	RU-1-8	33.44	4.37E-13
RU-1-0.5	RU-1-16	42.7	4.37E-13
RU-1-0.5	REG-0.5	32.07	4.37E-13
RU-1-0.5	COMB-0.5	9.382	5.16E-06
RU-1-1	RU-1-2	3.768	0.6072
RU-1-1	RU-1-4	4.142	0.4205
RU-1-1	RU-1-8	30.01	4.37E-13
RU-1-1	RU-1-16	39.26	4.37E-13
RU-1-1	REG-1	29.01	4.37E-13
RU-1-1	COMB-1	2.848	0.9497
RU-1-2	RU-1-4	7.91	0.0002218
RU-1-2	RU-1-8	33.77	4.37E-13
RU-1-2	RU-1-16	43.03	4.37E-13

RU-1-2	REG-2	18.07	4.65E-13
RU-1-2	COMB-2	15.95	8.37E-13
RU-1-4	RU-1-8	25.86	4.37E-13
RU-1-4	RU-1-16	35.12	4.37E-13
RU-1-4	REG-4	14.13	2.49E-11
RU-1-4	COMB-4	19.52	4.44E-13
RU-1-8	RU-1-16	9.255	7.17E-06
RU-1-8	REG-8	30.79	4.37E-13
RU-1-8	COMB-8	3.126	0.8833
RU-1-16	REG-16	28.44	4.37E-13
RU-1-16	COMB-16	1.974	0.9996
REG-0	REG-0.125	0.2776	1
REG-0	REG-0.25	1.943	0.9997
REG-0	REG-0.5	5.628	0.03997
REG-0	REG-1	12.13	3.96E-09
REG-0	REG-2	19.3	4.47E-13
REG-0	REG-4	31.15	4.37E-13
REG-0	REG-8	40.35	4.37E-13
REG-0	REG-16	51.96	4.37E-13
REG-0	COMB-0	0	1
REG-0.125	REG-0.25	1.666	1
REG-0.125	REG-0.5	5.35	0.06774
REG-0.125	REG-1	11.85	8.14E-09
REG-0.125	REG-2	19.02	4.47E-13
REG-0.125	REG-4	30.87	4.37E-13
REG-0.125	REG-8	40.08	4.37E-13
REG-0.125	REG-16	51.68	4.37E-13
REG-0.125	COMB-0.125	19.7	4.42E-13
REG-0.25	REG-0.5	3.684	0.6493
REG-0.25	REG-1	10.19	6.33E-07
REG-0.25	REG-2	17.36	4.93E-13
REG-0.25	REG-4	29.2	4.37E-13
REG-0.25	REG-8	38.41	4.37E-13
REG-0.25	REG-16	50.01	4.37E-13
REG-0.25	COMB-0.25	36.41	4.37E-13
REG-0.5	REG-1	6.502	0.006335
REG-0.5	REG-2	13.67	7.69E-11
REG-0.5	REG-4	25.52	4.37E-13
REG-0.5	REG-8	34.73	4.37E-13
REG-0.5	REG-16	46.33	4.37E-13
REG-0.5	COMB-0.5	41.45	4.37E-13
REG-1	REG-2	7.172	0.001343
REG-1	REG-4	19.02	4.46E-13
REG-1	REG-8	28.22	4.37E-13
REG-1	REG-16	39.83	4.37E-13
REG-1	COMB-1	31.85	4.37E-13

REG-2	REG-4	11.85	8.26E-09
REG-2	REG-8	21.05	4.39E-13
REG-2	REG-16	32.65	4.37E-13
REG-2	COMB-2	34.01	4.37E-13
REG-4	REG-8	9.207	8.13E-06
REG-4	REG-16	20.81	4.39E-13
REG-4	COMB-4	33.65	4.37E-13
REG-8	REG-16	11.6	1.56E-08
REG-8	COMB-8	33.91	4.37E-13
REG-16	COMB-16	30.41	4.37E-13
COMB-0	COMB-0.125	19.98	4.40E-13
COMB-0	COMB-0.25	38.35	4.37E-13
COMB-0	COMB-0.5	47.08	4.37E-13
COMB-0	COMB-1	43.98	4.37E-13
COMB-0	COMB-2	53.31	4.37E-13
COMB-0	COMB-4	64.8	4.37E-13
COMB-0	COMB-8	74.26	4.37E-13
COMB-0	COMB-16	82.37	4.37E-13
COMB-0.125	COMB-0.25	18.37	4.55E-13
COMB-0.125	COMB-0.5	27.09	4.37E-13
COMB-0.125	COMB-1	24	4.37E-13
COMB-0.125	COMB-2	33.33	4.37E-13
COMB-0.125	COMB-4	44.82	4.37E-13
COMB-0.125	COMB-8	54.28	4.37E-13
COMB-0.125	COMB-16	62.39	4.37E-13
COMB-0.25	COMB-0.5	8.727	2.81E-05
COMB-0.25	COMB-1	5.632	0.0396
COMB-0.25	COMB-2	14.97	3.67E-12
COMB-0.25	COMB-4	26.45	4.37E-13
COMB-0.25	COMB-8	35.92	4.37E-13
COMB-0.25	COMB-16	44.02	4.37E-13
COMB-0.5	COMB-1	3.095	0.8925
COMB-0.5	COMB-2	6.239	0.0113
COMB-0.5	COMB-4	17.72	4.72E-13
COMB-0.5	COMB-8	27.19	4.37E-13
COMB-0.5	COMB-16	35.29	4.37E-13
COMB-1	COMB-2	9.333	5.86E-06
COMB-1	COMB-4	20.82	4.39E-13
COMB-1	COMB-8	30.28	4.37E-13
COMB-1	COMB-16	38.39	4.37E-13
COMB-2	COMB-4	11.49	2.11E-08
COMB-2	COMB-8	20.95	4.39E-13
COMB-2	COMB-16	29.05	4.37E-13
COMB-4	COMB-8	9.464	4.17E-06
COMB-4	COMB-16	17.57	4.79E-13
COMB-8	COMB-16	8.104	0.0001366

FIXED-EFFECTS TWO-WAY ANOVA for IC50 values			IC50 TABLE		
	Sum of sqrs	df	Mean square	F	p (same)
Cell line:	37.6989	2	18.8495	484.8	2.22E-16
DRUG:	196.447	2	98.2233	2526	8.96E-23
Interaction:	48.3306	4	12.0826	310.7	2.43E-16
Within:	0.699881	18	0.0388823		
Total:	283.176	26			

	HCT116	REG-HCT116-R	HT29
HCT116		43.92	24.76
REG-HCT116-R	<0.0001		19.15
HT29	<0.0001	<0.0001	

	Ru-1(µM)	REG (µM)	REG/Ru-1 (µM)
Ru-1(µM)		85.5	3.028
REG (µM)	<0.0001		88.53
REG/Ru-1 (µM)	0.1218	<0.0001	
